# The Rare Codon AGA Is Involved in Regulation of Pyoluteorin Biosynthesis in *Pseudomonas protegens* Pf-5

**DOI:** 10.3389/fmicb.2016.00497

**Published:** 2016-04-19

**Authors:** Qing Yan, Benjamin Philmus, Cedar Hesse, Max Kohen, Jeff H. Chang, Joyce E. Loper

**Affiliations:** ^1^Department of Botany and Plant Pathology, Oregon State UniversityCorvallis, OR, USA; ^2^College of Pharmacy, Oregon State UniversityCorvallis, OR, USA; ^3^Horticultural Crops Research Laboratory, US Department of Agriculture, Agricultural Research ServiceCorvallis, OR, USA

**Keywords:** *Pseudomonas protegens*, rare codon, AGA codon, pyoluteorin, regulation

## Abstract

The soil bacterium *Pseudomonas protegens* Pf-5 can colonize root and seed surfaces of many plants, protecting them from infection by plant pathogenic fungi and oomycetes. The capacity to suppress disease is attributed to Pf-5's production of a large spectrum of antibiotics, which is controlled by complex regulatory circuits operating at the transcriptional and post-transcriptional levels. In this study, we analyzed the genomic sequence of Pf-5 for codon usage patterns and observed that the six rarest codons in the genome are present in all seven known antibiotic biosynthesis gene clusters. In particular, there is an abundance of rare codons in *pltR*, which encodes a member of the LysR transcriptional regulator family that controls the expression of pyoluteorin biosynthetic genes. To test the hypothesis that rare codons in *pltR* influence pyoluteorin production, we generated a derivative of Pf-5 in which 23 types of rare codons in *pltR* were substituted with synonymous preferred codons. The resultant mutant produced pyoluteorin at levels 15 times higher than that of the wild-type Pf-5. Accordingly, the promoter activity of the pyoluteorin biosynthetic gene *pltL* was 20 times higher in the codon-modified stain than in the wild-type. *pltR* has six AGA codons, which is the rarest codon in the Pf-5 genome. Substitution of all six AGA codons with preferred Arg codons resulted in a variant of *pltR* that conferred increased pyoluteorin production and *pltL* promoter activity. Furthermore, overexpression of tRNA${}_{\text{UCU}}^{\text{Arg}}$, the cognate tRNA for the AGA codon, significantly increased pyoluteorin production by Pf-5. A bias in codon usage has been linked to the regulation of many phenotypes in eukaryotes and prokaryotes but, to our knowledge, this is the first example of the role of a rare codon in the regulation of antibiotic production by a Gram-negative bacterium.

## Introduction

Intensive efforts have been made in the past decades to elucidate the regulatory mechanisms of antibiotic biosynthesis because of the important roles of antibiotics in agriculture and medicine. In bacteria, genes responsible for biosynthesis, efflux, and regulation of an antibiotic are typically grouped together in the genome as gene clusters. Expression of antibiotic biosynthetic genes is often tightly controlled at transcriptional and posttranscriptional levels by pathway-specific regulators, which commonly are encoded by genes located within the biosynthetic gene cluster, and global regulators encoded by genes distributed distally in the genome (Gross and Loper, 2009).

Biased usage of codons is a well-established mechanism for the post-transcriptional regulation of gene expression in bacteria (as reviewed in Ling et al., 2015; Quax et al., 2015). The genetic code is degenerate: 18 of the 20 standard amino acids are encoded by multiple synonymous codons and the usage frequencies of synonymous codons within a genome differ from one another (Chaney and Clark, 2015; Ling et al., 2015). Codon usage is thought to influence protein expression and activity through a variety of mechanisms, including alterations in translational rate and accuracy, co-translational protein folding and interaction with other cellular components during translation, and the stability of mRNA structure (Subramaniam et al., 2013; Chaney and Clark, 2015; Ling et al., 2015; Quax et al., 2015; Rahmen et al., 2015; Supek, 2016). In many organisms, strongly-expressed genes have more common codons than do weakly-expressed genes, and the cognate tRNAs for common codons are usually at relatively high abundance in the cell (Ikemura, 1985; Elf et al., 2003). Compared with the more commonly-used codons, rare synonymous codons are generally believed to be translated more slowly due to the lower level of cognate tRNA (Sørensen et al., 1989; Letzring et al., 2010). Biased codon usage is involved in diverse microbial functions including photosynthesis (Peden, 1999), stress response (Chan et al., 2012), colony differentiation (Nguyen et al., 2003), lipopolysaccharide synthesis (Daniels et al., 1998), expression of integrase proteins involved in bacteriophage DNA integration and excision (Zahn and Landy, 1996), and expression of type I fimbriae (Tinker and Clegg, 2001). UUA, the rarest codon in the genome of the Gram-positive bacterium *Streptomyces coelicolor*, is present in several antibiotic biosynthesis gene clusters and has a well-established role in the regulation of antibiotic production (Chandra and Chater, 2008). A mutation in *bldA*, which encodes the cognate tRNA for the UUA codon in *S. coelicolor* (Lawlor et al., 1987), has no obvious effect on vegetative growth but abolishes expression of antibiotic biosynthetic genes and the production of antibiotics such as actinorhodin, undecylprodigiosin, methylenomycin, and a calcium-dependent antibiotic (Guthrie and Chater, 1990). The dependence of antibiotic production on *bldA* can be relieved by substituting the UUA rare codon with a synonymous codon in genes regulating the biosynthesis of these antibiotics. For example, the biosynthesis of actinorhodin requires the pathway-specific activator gene *actII*-ORF4, which contains the UUA rare codon. Substitution of the UUA codon with the synonymous UUG codon in *actII*-ORF4 allows actinorhodin biosynthesis in *bldA* mutants (Fernández-Moreno et al., 1991). To date, however, the role of codon usage in regulation of secondary metabolism in Gram-negative bacteria is virtually unknown.

In this work, we investigated the role of codon usage in antibiotic biosynthesis by *Pseudomonas protegens* strain Pf-5. This soil bacterium is a model organism for molecular studies of secondary metabolites with anti-microbial activity because of the large spectrum of antibiotics characterized in this strain (Paulsen et al., 2005; Loper and Gross, 2007; Gross and Loper, 2009). The seven known antibiotics produced by Pf-5 are pyrrolnitrin (Howell and Stipanovic, 1979), hydrogen cyanide (HCN) (Kraus and Loper, 1992), 2,4-diacetylphloroglucinol (DAPG) (Nowak-Thompson et al., 1994), pyoluteorin (Howell and Stipanovic, 1980; Nowak-Thompson et al., 1999), orfamide A (Gross et al., 2007), rhizoxin analogs (Brendel et al., 2007; Loper et al., 2008), and toxoflavin (Philmus et al., 2015). In our ongoing investigations, we noticed that rare codons are present in many antibiotic biosynthesis gene clusters of Pf-5 and hypothesized that rare codon(s) play a role in antibiotic production by strain Pf-5. Here we report that AGA, the rarest codon in the Pf-5 genome, is involved in the regulation of pyoluteorin production of Pf-5. The results of this study highlight the importance of codon usage in regulation of antibiotic production in a Gram- negative bacterium.

## Materials and methods

### Strains and cultural conditions

The bacterial strains used in this study are listed in Table 1. *Pseudomonas protegens* Pf-5 and mutant strains were cultured at 27°C on King's Medium B agar (King et al., 1954), Nutrient Agar (Becton, Dickinson and Company, Sparks, MD) supplemented with 1% glycerol (NAGly) or Nutrient Broth (Becton, Dickenson and Company) supplemented with 1% glycerol (NBGly). Liquid cultures were grown with shaking at 200 r.p.m.

**Table 1:** **Bacterial strains, plasmids and primers used in this study**.

**Strains, plasmids, or primers**	**Genotype, relevant characteristics, or sequences**	**References or source**
**STRAINS**		
***P. protegens***		
LK099	Wild-type strain Pf-5	Howell and Stipanovic, 1979
LK298	Pf-5 derivative strain contains *pltR* with modifications in 35 types of rare codons in the chromosome	This study
LK361	Pf-5 derivative strain contains *pltR* with modifications in six types of rare codons in the chromosome. The modified codons are CUA, GUU, AUA, CUU, UAU, and GUA	This study
LK362	Pf-5 derivative strain contains *pltR* with modifications in six types of rare codons in the chromosome. The modified codons are ACU, GGU, GGA, GCU, CCA, and CCU	This study
LK363	Pf-5 derivative strain contains *pltR* with modifications in six types of rare codons in the chromosome. The modified codons are UCU, UGU, CGA, UUG, UUU, and GCA	This study
LK364	Pf-5 derivative strain contains *pltR* with modifications in five types of rare codons in the chromosome. The modified codons are UUA, AUU, AGU, AGG, and AGA	This study
LK365	Pf-5 derivative strain contains *pltR* with modifications in the AGA rare codon in the chromosome	This study
***E. coli***		
Top10	F- mcrA Δ(*mrr-hsdRMS-mcrBC*) Φ80*lacZ*ΔM15 Δ*lacX*74 *recA*1 *araD*139 Δ(*ara-leu*)7697 *galU galK rpsL* (Sm^r^) *endA*1 *nupG*	Invitrogen
S17-1	*recA pro hsdR*^−^M^+^ RP4 2-Tc::Mu-Km::Tn7 Sm^r^ Tp^r^	Simon et al., 1983
**PLASMIDS**		
pEX18Km	Gene replacement vector with MCS from pUC18, *sacB*^+^ Km^r^	Hoang et al., 1998
pEX18km-pltR-MCod3	pEX18Km with a 1.6 Kb *Xba*I fragment synthesized from IDT, containing *pltR* of Pf-5 with modifications in 35 types of codons	This study
pEX18Tc	Gene replacement vector with MCS from pUC18, sacB^+^ Tc^r^	Hoang et al., 1998
pEX18Tc-pltR-Mcod4-1	pEX18Tc with a 1.6 Kb *Xba*I fragment synthesized from IDT, containing *pltR* of Pf-5 with modifications in six types of codons (CUA, GUU, AUA, CUU, UAU and GUA).	This study
pEX18Tc-pltR-Mcod4-2	pEX18Tc with a 1.6 Kb *Xba*I fragment synthesized from IDT, containing *pltR* of Pf-5 with modifications in six types of codons (ACU, GGU, GGA, GCU, CCA, and CCU).	This study
pEX18Tc-pltR-Mcod4-3	pEX18Tc with a 1.6 Kb *Xba*I fragment synthesized from IDT, containing *pltR* of Pf-5 with modifications in six types of codons (UCU, UGU, CGA, UUG, UUU and GCA).	This study
pEX18Tc-pltR-Mcod4-4	pEX18Tc with a 1.6 Kb *Xba*I fragment synthesized from IDT, containing *pltR* of Pf-5 with modifications in five types of codons (UUA, AUU, AGU, AGG and AGA).	This study
pEX18Tc-pltR-Mcod5	pEX18Tc with a 1.6 Kb *Xba*I fragment synthesized from IDT, containing *pltR* of Pf-5 with modifications in the AGA rare codons.	This study
pPROBE'-gfp(tagless)	pBBR1, containing promoterless *gfp*, Km^r^	Miller et al., 2000
pprnA-gfp (AGA)	pPROBE'-gfp(tagless) with a 1.3 Kb *Hin*dIII-*Sal*I PCR fragment amplified from genomic DNA of Pf-5, containing a *prnA::gfp* (AGA) transcriptional fusion	This study
pprnA-gfp (CGC)	pprnA-gfp withthe AGA codons of *gfp* were substituted with synonymous common codons, containing a *prnA::gfp*(CGC) transcriptional fusion	This study
ppltL-gfp	pPROBE'-gfp(tagless) with a 0.1 Kb *Eco*RI-*Kpn*I PCR fragment amplified from genomic DNA of Pf-5, containing a *pltL::gfp* transcriptional fusion	This study
pME6010	pACYC177-pVS1 shuttle vector, Tc^r^	Heeb et al., 2000
P6010-Arg	pME6010 with a 0.6 Kb *Sal*I-*Eco*RI PCR fragment amplified from the genomic DNA of Pf-5, containing a constitutively expressed PFL_3991	This study
**Primers (5′–3′)**		
prnA-f2	TATAAGCTTTGCCGCCATGGCCAACGCC	
prnA-r1	CAAAGATGCAGTAGTAGTTGCCG	
gfp-plt-f3	ATAGAATTCGGGGCTGTTTTGCCTTTGC	
gfp-plt-r1	ATGGTACCATAGACGTACGCTCCTGC	
3989-91-DnR-SalI	ATAGTCGACTTGAGTGGTGTTGGCGGGCA	
3991-R2	ATAGAATTCTTGTGCCAAAGCTGCCT	

### Modification of rare codons with common synonymous codons

To modify the rare codons of *pltR* in the chromosome of Pf-5, six 1632-bp DNA fragments containing *pltR* with different substitutions of synonymous codons (**Table 3**) and the 300 bp flanking *pltR* in the Pf-5 genome were synthesized at Integrated DNA Technologies (IDT, Coralville, Iowa, USA). The synthesized DNA fragment was ligated into pEX18Km or pEX18Tc. The resultant plasmid were transformed into *E. coli* S17-1. Derivative strains of Pf-5 having a codon-modified *pltR* in the chromosome were obtained using an allelic exchange strategy described previously (Kidarsa et al., 2011). The nucleotide sequences of codon-modified *pltR* genes in LK298, LK361, LK362, LK363, LK364, and LK365 were confirmed by sequencing PCR amplicons from each strain, and are shown in Supplemental file 1.

The *gfp* gene in plasmid pPROBE'-gfp(tagless) contains five copies of the AGA rare codon (Miller et al., 2000). An 829-bp DNA fragment that contains a modified *gfp* with all five AGA codons substituted with CGC, a synonymous common codon, was synthesized at IDT. The synthesized DNA fragment also contained a *Hin*dIII restriction enzyme site at both ends of the *gfp* sequence. The plasmid pPROBE'-gfp(tagless) was digested with *Hin*dIII to remove the unmodified *gfp*, and the digested vector was ligated to the synthesized DNA fragment containing the codon-modified *gfp*, which had also been digested with *Hin*dIII. The resultant plasmid pPROBE'-gfp(CGC) was sequenced to confirm the modification of the AGA codons and the orientation of *gfp*. The promoter of *prnA* was amplified with primers prnA-f2 and prnA-r1 (Table 1). The PCR product was digested with *Hin*dIII and *Sal*I, and the resultant 1361-bp fragment was ligated into pPROBE'-gfp(tagless) and pPROBE'-gfp(CGC) to generate the transcriptional fusions *prnA::gfp*(AGA) and *prnA::gfp*(CGC), respectively. The plasmids were sequenced to confirm that both contain the same promoter sequence upstream of *gfp*.

### Construction of the *pltL::gfp* transcriptional fusion

A region of the Pf-5 genome encompassing the putative binding site of PltR and the promoter region of *pltL* was amplified by the PCR with the primer pair gfp-plt-f3 and gfp-plt-r1 (Table 1). The resultant 134-bp PCR amplicon was digested with *Eco*RI and *Kpn*I and ligated into pPROBE'-gfp(tagless) to generate the promoter fusion *pltL::gfp*. The resultant plasmid ppltL-gfp was introduced into wild-type Pf-5 and its derivative strains using a bi-parental mating method (Kidarsa et al., 2011).

### Overexpression of the tRNA${}_{\text{UCU}}^{\text{Arg}}$ in Pf-5

PFL_3991, which encodes tRNA${}_{\text{UCU}}^{\text{Arg}}$, was amplified by the PCR with primers 3989-91-DnR-SalI and 3991-R2 (Table 1) by using genomic DNA of Pf-5 as the template. The 605-bp amplicon was digested with *Sal*I and *Eco*RI and ligated to pME6010, placing PFL_3991 under the control of a constitutive kanamycin-resistance promoter (Pk) (Heeb et al., 2000), which has been used to overexpress other genes of interest (Manuel et al., 2011; Zhang et al., 2012). The 605-bp insert in the resultant plasmid, p6010-Arg, was confirmed to be as expected by sequencing, and the plasmid was introduced into wild-type Pf-5 by bi-parental mating.

### Assays for monitoring GFP expression

The Pf-5 strains containing *gfp*-based reporter plasmids were cultured overnight in NBGly at 27°C with shaking at 200 r.p.m. The cells were washed once with NBGly and used to inoculate 200 μl NBGly to obtain an optical density at 600 nm (OD_600_) of 0.01. Each strain was grown in three wells of a 96-well plate, which was incubated in a 96-well plate reader (Tecan Infinite 200Pro, Männedorf, Switzerland) at 27°C with shaking at approximately 200 r.p.m. Growth of the bacteria was monitored by measuring the OD_600_. The green fluorescence of bacteria was monitored by measuring emission at 535 nm with an excitation at 485 nm and corrected for background by subtracting fluorescence emitted by the growth medium.

### Antibiotic extraction and quantification

Pf-5 and derivative strains were cultured overnight in 5 ml NBGly at 27°C. The cells were washed once with fresh NBGly, suspended in sterile NBGly, and bacterial suspensions were used to inoculate triplicate culture tubes containing 5 ml NBGly to an OD_600_ of 0.01. Bacteria were cultured for 24 h at 27°C with shaking (200 r.p.m.). One milliliter of the culture was used to measure the OD_600_. Four milliliters of the culture were extracted twice with 2.5 ml ethyl acetate. The ethyl acetate extracts were dried under vacuum and suspended in 100 μl methanol and a portion (10 μl) was analyzed by High-pressure liquid chromatography (HPLC). HPLC analyses were accomplished using an Agilent 1100 HPLC instrument, which consisted of a quaternary pump, vacuum degasser, autosampler, column thermostat (set to 30°C), and diode array detector. Separation was achieved using a Luna C18 column (4.6 × 150 mm, 5 μm, Phenomenex, Torrance, CA) with a flow rate of 1 ml/min where line A was water + 0.1% (vol/vol) formic acid, and line B was methanol + 0.1% (vol/vol) formic acid with the following program. The column was pre-equilibrated in 90% A/10% B and upon injection this composition was held for 2 min. The composition of mobile phase was then changed to 0% A/100% B over 28 min utilizing a linear gradient. This composition was held for 6 min followed by changing to 90% A/10% B over 2 min. The column was equilibrated in 90% A/10% B for 6 min prior to the next injection. Under these chromatographic conditions, pyoluteorin eluted at 15.1 min. The HPLC was operated with and data was viewed using ChemStation (version B.04.03, Agilent, Santa Clara, CA). Quantification was performed by integrating the area under the curve at 300 nm and comparing to a standard curve prepared by injection of purified pyoluteorin. Data was processed with GraphPad Prism (GraphPad Software, San Diego, CA).

## Results

### Rare codons are present in the antibiotic biosynthesis gene clusters of *P. protegens* Pf-5

**Figure 1:**
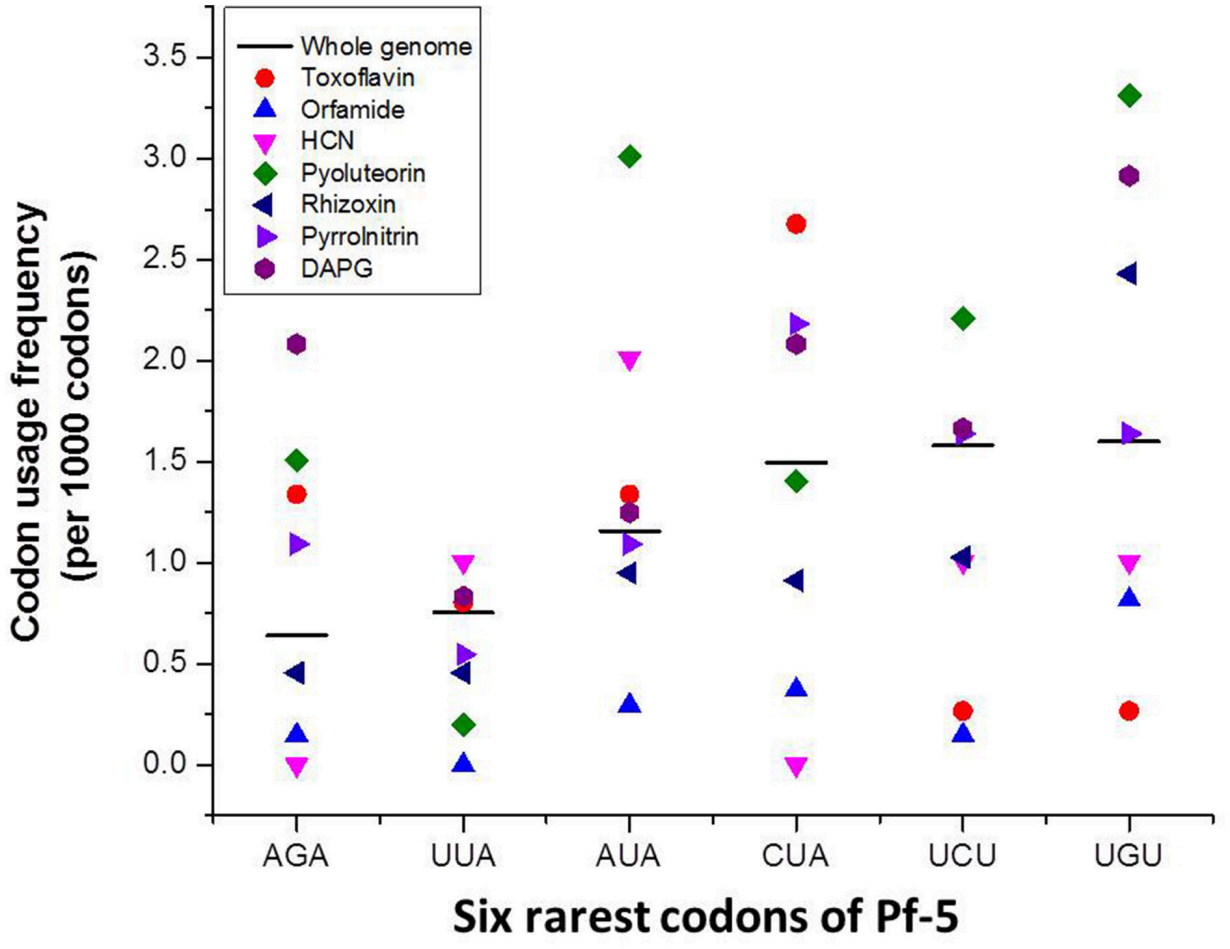
**The codon usage frequency of the six rarest codons in antibiotic gene clusters of *P. protegens* Pf-5**. The codon usage frequency refers to the number of times a specified codon was identified per 1000 codons in the annotated coding sequences of Pf-5 (black bar) or within a specific gene cluster (colored markers).

The arginine (Arg) codon AGA is the rarest codon in the annotated coding sequences (CDSs) of the Pf-5 genome, occurring at the level of 9.9 per 1000 Arg codons and 0.6 per 1000 codons (Table 2; Paulsen et al., 2005). AGA is present in 917 genes, which comprise approximately 15% of the 6144 annotated genes in the Pf-5 genome (Table 2). Six of the seven antibiotic biosynthesis gene clusters of Pf-5 include at least one AGA codon and 40% of the genes present in the seven gene clusters have an AGA codon (Table 2). The usage frequency of the AGA codon within four antibiotic biosynthesis gene clusters exceeds the average usage frequency of this rare codon in the genome of Pf-5 (Figure 1). Further, analysis revealed that each of the top six rarest codons in the Pf-5 genome is present in the six or more of the seven antibiotic biosynthesis gene clusters (Table 2). Fifty five of the 60 genes (92%) in the seven gene clusters have at least one of the six rarest codons of the Pf-5 genome (Table 2). The presence of rare codons in the antibiotic gene clusters of Pf-5 raised the possibility that rare codons may be involved in the regulation of antibiotic production in this bacterium.

**Table 2:** **Summary of the six rarest codons in the Pf-5 genome**.

**Codon**	**Amino acid**	**Codon usage frequency (per 1000 codons)**		**Total genome (6144 genes)**		**Toxoflavin (PFL_1028–1037)**		**Orfamide A (PFL_2142–2150)**		**HCN (PFL_2577–2579)**		**Pyoluteorin (PFL_2784–2800)**		**Rhizoxin (PFL_2989–2997)**		**Pyrrolnitrin (PFL_3604–3607)**		**DAPG (PFL_5951–5958)**		**All seven clusters (60 genes)**	
		**Of total codons**	**Of total synonymous codons**	**# CDS**	**%**	**# CDS**	**%**	**# CDS**	**%**	**# CDS**	**%**	**# CDS**	**%**	**# CDS**	**%**	**# CDS**	**%**	**# CDS**	**%**	**# CDS**	**%**
AGA	Arg	0.6	9.9	917	15	4	40	2	22	0	0	7	41	5	56	2	50	4	50	24	40
UUA	Leu	0.8	6.2	1049	17	2	20	0	0	1	33	2	12	6	67	1	25	2	25	14	23
AUA	Ile	1.2	25.2	1547	25	3	30	3	33	2	67	14	82	9	100	2	50	3	38	36	60
CUA	Leu	1.5	12.3	1967	32	2	20	2	22	0	0	7	41	7	78	2	50	4	50	24	40
UCU	Ser	1.6	77.8	2052	33	1	10	2	22	1	33	10	59	7	78	2	50	3	38	26	43
UGU	Cys	1.6	154.0	2226	36	1	10	7	78	1	33	11	65	9	100	3	75	4	50	36	60

*^#^CDS means coding sequences. % indicates the percentage of rare codon-containing genes in the total genome of Pf-5 or in each antibiotic gene cluster*.

### Synonymous substitutions of rare codons in *pltR* increase pyoluteorin production

**Figure 2:**
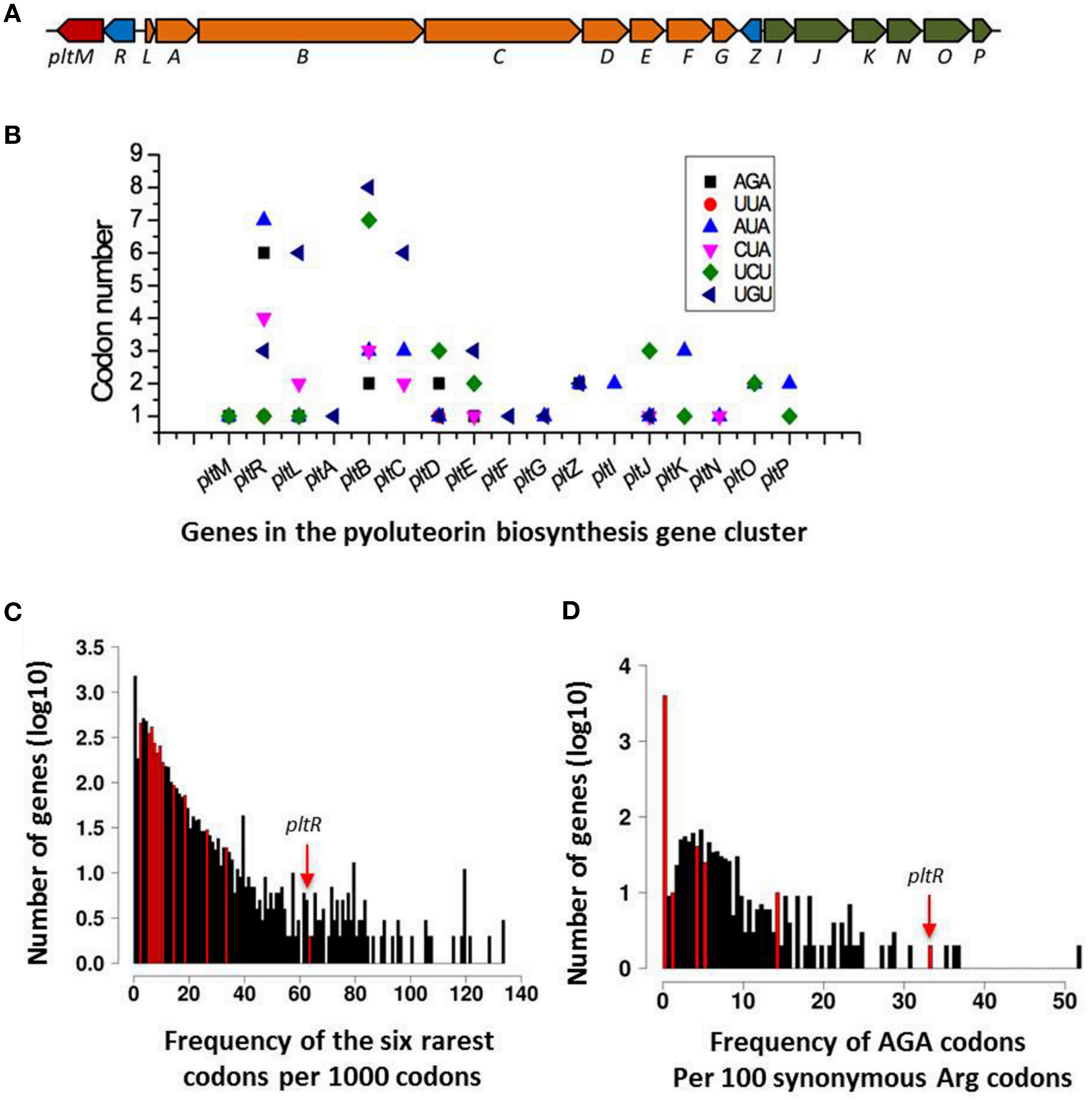
**Distribution of rare codons in the pyoluteorin gene cluster of *P. protegens* Pf-5**. **(A)** Diagram of the pyoluteorin gene cluster of Pf-5. Colors denote genes that function in biosynthesis (orange), transport (blue), regulation (blue), or other processes (red) associated with the production of pyoluteorin. **(B)** Distribution of Pf-5's six rarest codons within the pyoluteorin gene cluster. **(C)** Frequency histogram depicting the relative abundance of rare codons for each gene in the Pf-5 genome. The six rarest codons in the Pf-5 genome are normalized to rare codons per 1000 codons and plotted in bins on the X-axis. Y-axis is the log(10)-transformed number of genes in each bin. Bins containing genes within the pyoluteorin gene cluster are colored red. Arrow shows the bin containing the *pltR* gene. **(D)** Frequency histogram of the AGA codon usage relative to all synonymous arginine codons for each gene in the Pf-5 genome. Relative AGA usage is presented as percent AGA encoded arginine along the X-axis with log(10)-transformed abundances along the Y-axis. Only genes containing at least five arginine codons were included in this plot. Bins containing genes within the pyoluteorin gene cluster are highlighted in red. Arrow shows the bin containing *pltR* gene.

To test the hypothesis that rare codons play a role in the regulation of antibiotic production, we chose to focus on the pyoluteorin biosynthesis gene (*plt*) cluster because it has a large number of rare codons (Figure 2) and has been the subject of intensive study by our group (Nowak-Thompson et al., 1999; Brodhagen et al., 2004, 2005; Kidarsa et al., 2011). The *plt* cluster consists of genes with regulatory, biosynthetic, and transport functions (Figure 2A; Nowak-Thompson et al., 1999). It has been noted that *pltR*, which encodes a LysR family regulator required for transcriptional activation of pyoluteorin biosynthetic genes, has a higher frequency of rare codons than do the other genes in the *plt* gene cluster (Figure 2C; Nowak-Thompson et al., 1999). *pltR* contains all 61 amino acid-encoding codons except CGT, a preferred Arg codon with a usage frequency of 9.2 codons per 1000 codons (Table 3). To determine if the rare codons in *pltR* influence pyoluteorin production, we made a derivative of Pf-5 having a modified *pltR* in which 35 different types of rare codons were substituted with preferred synonymous codons, resulting in alterations to 126 of the 343 codons in *pltR* (Figure 3A). Among the 35 optimized codons, 23 were completely substituted (Table 3). 12 codons were partially substituted (Table 3) because complete codon modification was predicted to result in complex secondary structures of DNA oligonucleotides that were difficult to synthesize. The resultant codon-modified strain, LK298, produced 15 times more pyoluteorin than did the wild-type strain Pf-5 (Figure 3B). The final cell density of the 24 h culture of LK298 was slightly lower than the wild-type (Figure 3B), which is consistent with our previous observation of an inverse relationship between the amount of pyoluteorin produced in a Pf-5 culture and the culture's final cell density (Kidarsa et al., 2011). However, the slight growth difference did not affect the result that codon optimization in *pltR* promoted pyoluteorin production.

**Table 3:** **Codon usage of *pltR* gene in Pf-5 and the codon-modified p*ltR* derivatives**.

**Amino Acid**	**Codon**	**Frequency(/1000)^#^**	**Numbers of codon in *pltR***						
			**WT**	**LK298**	**LK361**	**LK362**	**LK363**	**LK364**	**LK365**
Ala	GCA	7.8	7	0	7	7	0	7	7
Ala	GCU	7.8	2	0	2	0	2	2	2
Ala	GCG	28.4	7	8	7	7	11	7	7
Ala	GCC	67.4	7	15	7	9	10	7	7
Arg	AGA	0.6	6	0	6	6	6	0	0
Arg	AGG	1.6	1	0	1	1	1	0	1
Arg	CGA	2.3	1	0	1	1	0	1	1
Arg	CGU	9.2	0	2	0	0	0	0	0
Arg	CGG	14.2	4	3	4	4	4	6	6
Arg	CGC	36.6	6	13	6	6	7	11	10
Asn	AAU	5.9	5	1	5	5	5	5	5
Asn	AAC	23.6	6	10	6	6	6	6	6
Asp	GAU	14.9	6	3	6	6	6	6	6
Asp	GAC	20.6	10	13	10	10	10	10	10
Cys	UGU	1.6	3	0	3	3	0	3	3
Cys	UGC	8.8	6	9	6	6	9	6	6
End	UAG	0.4	0	0	0	0	0	0	0
End	UAA	0.7	0	0	0	0	0	0	0
End	UGA	1.9	1	1	1	1	1	1	1
Gln	CAA	10.3	7	1	7	7	7	7	7
Gln	CAG	40.4	5	11	5	5	5	5	5
Glu	GAA	26.5	15	8	15	15	15	15	15
Glu	GAG	28	3	10	3	3	3	3	3
Gly	GGA	2.7	4	0	4	0	4	4	4
Gly	GGU	11.4	2	0	2	0	2	2	2
Gly	GGG	13.7	1	1	1	2	1	1	1
Gly	GGC	52.7	11	17	11	16	11	11	11
His	CAU	7	9	4	9	9	9	9	9
His	CAC	15.9	4	9	4	4	4	4	4
Ile	AUA	1.2	7	0	0	7	7	7	7
Ile	AUU	7.6	3	0	3	3	3	0	3
Ile	AUC	37.2	14	24	21	14	14	17	14
Leu	UUA	0.8	1	0	1	1	1	0	1
Leu	CUA	1.5	4	0	0	4	4	4	4
Leu	CUU	3.8	10	0	0	10	10	10	10
Leu	UUG	13.2	8	0	8	8	0	8	8
Leu	CUC	16.5	9	18	15	9	10	10	9
Leu	CUG	86.2	16	30	24	16	23	16	16
Lys	AAA	7	8	3	8	8	8	8	8
Lys	AAG	24.8	10	15	10	10	10	10	10
Met	AUG	21.4	5	5	5	5	5	5	5
Phe	UUU	7.1	3	0	3	3	0	3	3
Phe	UUC	28.7	7	10	7	7	10	7	7
Pro	CCA	4.1	2	0	2	0	2	2	2
Pro	CCU	4.1	3	0	3	0	3	3	3
Pro	CCC	16.3	5	2	5	7	5	5	5
Pro	CCG	25.7	7	15	7	10	7	7	7
Ser	UCU	1.6	1	0	1	1	0	1	1
Ser	UCA	1.7	5	1	5	5	5	5	5
Ser	AGU	4.4	3	0	3	3	3	0	3
Ser	UCG	8.8	8	7	8	8	8	8	8
Ser	UCC	12.7	4	8	4	4	4	6	4
Ser	AGC	27.5	13	18	13	13	14	14	13
Thr	ACA	1.7	4	1	4	4	4	4	4
Thr	ACU	3.6	3	0	3	0	3	3	3
Thr	ACG	5.6	3	2	3	3	3	3	3
Thr	ACC	34.7	12	19	12	15	12	12	12
Trp	UGG	14.7	3	3	3	3	3	3	3
Tyr	UAU	6.6	2	0	0	2	2	2	2
Tyr	UAC	18.8	4	6	6	4	4	4	4
Val	GUU	4.3	4	0	0	4	4	4	4
Val	GUA	4.6	3	0	0	3	3	3	3
Val	GUC	20.6	5	10	8	5	5	5	5
Val	GUG	38.8	6	8	10	6	6	6	6

# *Frequency indicates the codon usage frequency of a specific codon per 1000 total codons used in the annotated coding sequences of Pf-5*.

**Figure 3:**
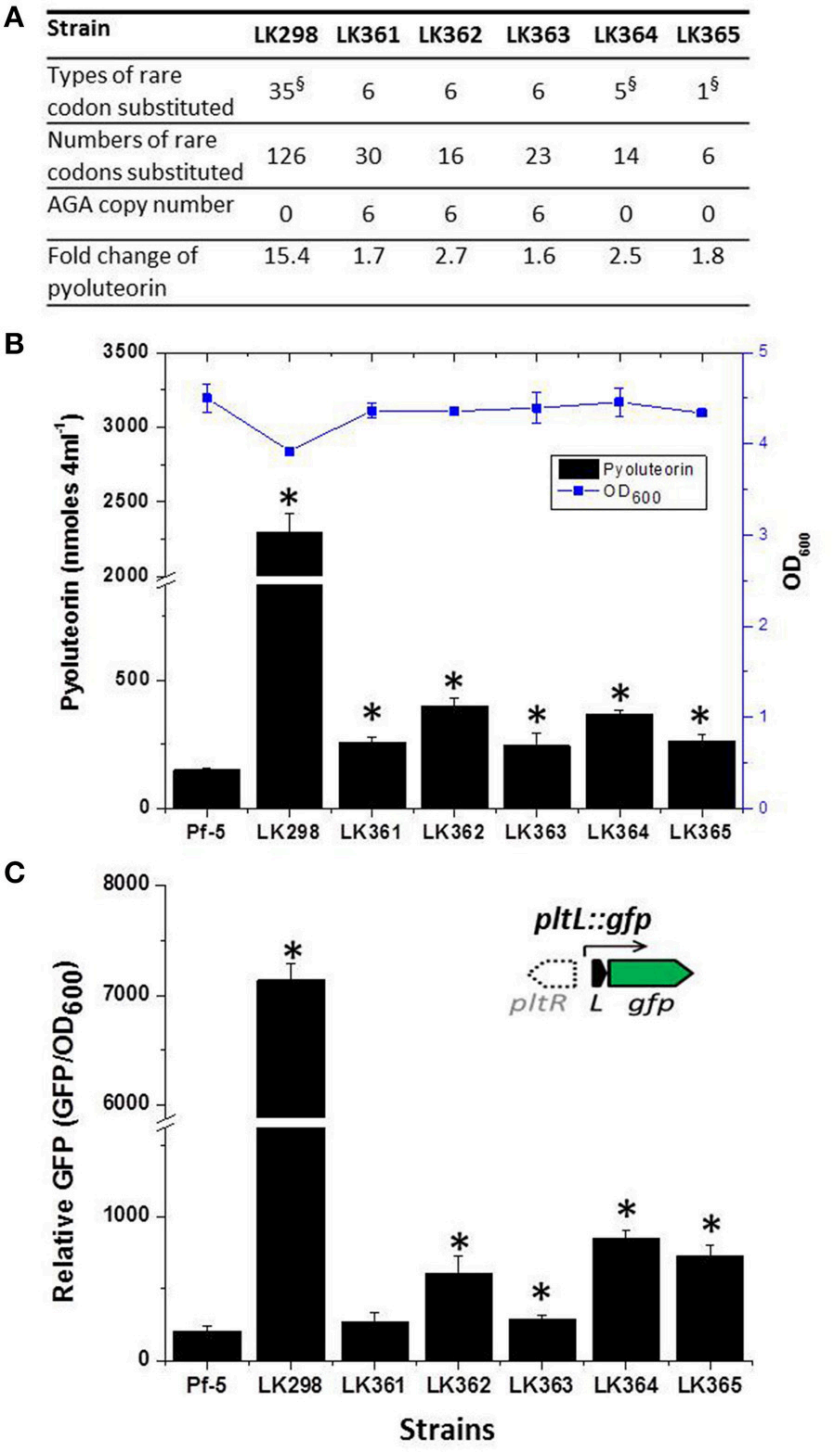
**Substitutions of rare codons of *pltR* with preferred synonymous codons increase pyoluteorin production and transcription of the pyoluteorin biosynthetic gene *pltL***. **(A)** The number of types of codons and the number of total codons modified in each strain. Detailed information on the specific substitutions in *pltR* for each strain is provided in Table 3. ^§^ indicates the substituted codons of these strains include AGA, which occurs six times in *pltR* of Pf-5. Four AGA codons were replaced with CGC and two were replaced with CGG in the modified *pltR* genes of LK298, LK364, and LK365. Fold change refers to the concentration of pyoluteorin produced by each strain relative to wild-type Pf-5 as shown in **(B)**. **(B)** Production of pyoluteorin by wild-type Pf-5 and derivative strains having *pltR* genes with modifications in specific codons. The antibiotic production (black bars) and the growth (OD_600_) (blue line) of each strain are shown. Values represent the average of at least three replicates and error bars show the standard deviation. Asterisks denote strains that produce levels of pyoluteorin that differ significantly from that of wild-type strain Pf-5 by a student *t*-test analysis (*p* < 0.01). **(C)**. Substitution of the AGA rare codon with common synonymous codons of *pltR* increased the promoter activity of *pltL* assessed with a *pltL::gfp* transcriptional fusion. Inset, the location of *pltR* (open arrow) relative to *pltL* is shown for reference, but *pltR* was not included in the construct. The promoter activity of *pltL* in derivatives of Pf-5 containing the wild-type and the codon-modified *pltR* genes and the *pltL::gfp* transcriptional fusion was assessed by measuring GFP fluorescence normalized by growth (OD_600_); Values represent the average of three replicates and error bars show the standard deviation. Asterisks denote strains in which the promoter activity of *pltL* is significantly higher than in wild-type Pf-5, as determined by a Student's *t*-test (*p* < 0.05).

### Substituting the rare codon AGA with a preferred synonymous codon in *pltR* increases pyoluteorin production

To pinpoint the rare codon(s) important in the *pltR*-mediated regulation of pyoluteorin production, we created another four Pf-5 derivatives, each containing a variant of *pltR* having five to six substitutions of rare codons that were modified in LK298 with preferred synonymous codons (Figure 3A, Table 3). Collectively, the four derivative strains (LK361, LK362, LK363, and LK364) had substitutions in all 23 codons that were completely substituted in LK298. Quantification of pyoluteorin by HPLC indicated that all four derivative strains produced significantly higher amounts of pyoluteorin relative to wild-type Pf-5 (Figure 3B).

Among the modified codons of the *pltR*-derivative strains, the AGA codon drew our interest because AGA is the rarest codon in strain Pf-5, is overrepresented in *pltR* (Figure 2D), and was substituted with preferred synonymous codons in both LK298 and LK364, which overproduce pyoluteorin (Figure 3B). Therefore, we made a Pf-5 derivative strain (LK365) in which only the AGA rare codon (six copies) was substituted with preferred synonymous codons in *pltR*. Quantification of pyoluteorin showed that LK365 produced more pyoluteorin than the wild-type (Figure 3B). Thus, our result indicated that substituting the AGA rare codon of *pltR* with a synonymous preferred codon increased pyoluteorin production in Pf-5.

### Overexpression of the gene encoding tRNA${}_{\text{UCU}}^{\text{Arg}}$ increases pyoluteorin production

**Figure 4:**
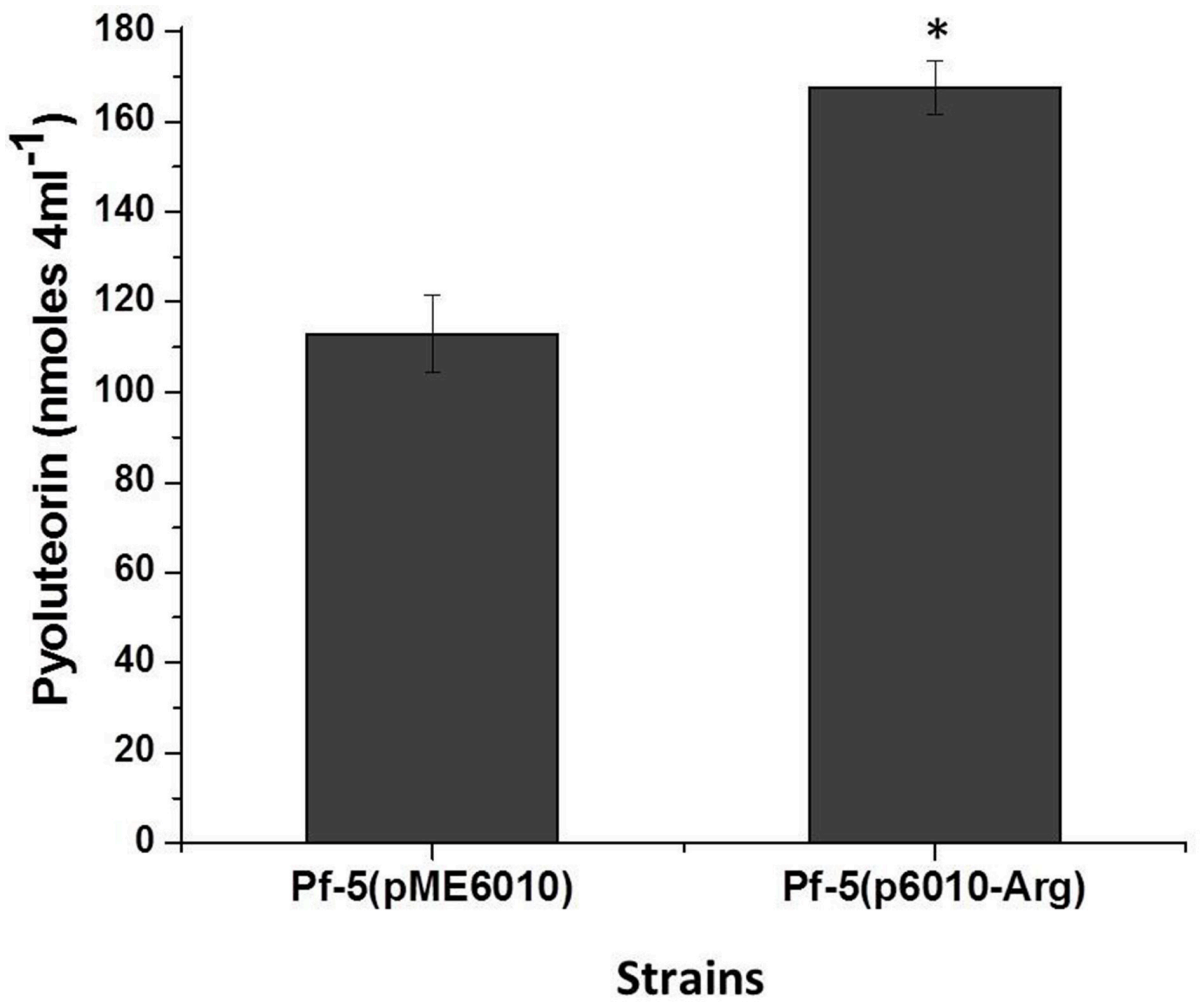
**Overexpression of the tRNA${}_{\text{UCU}}^{\text{Arg}}$ encoding gene PFL_3991 increased pyoluteorin production by *P. protegens* Pf-5**. PFL_3991 was expressed from a constitutive promoter in plasmid p6010-Arg. Pyoluteorin production of the wild-type strain containing p6010-Arg, denoted with an asterisk, was significantly higher (student *t*-test, *p* < 0.01) than the wild-type containing the empty vector pME6010. Values represent the average of three replicates and error bars show the standard deviation.

We have shown that the codon AGA in *pltR* is involved in pyoluteorin production, so it is reasonable to propose that the abundance of the cognate tRNA deciphering the AGA codon will influence the *pltR*-mediated regulation of pyoluteorin production. Of the five genes encoding tRNA^Arg^ in Pf-5, PFL_3991 encodes the tRNA${}_{\text{UCU}}^{\text{Arg}}$ deciphering the AGA rare codon. Our repeated efforts to delete PFL_3991 from the chromosome of Pf-5 failed (data not shown), possibly due to an essential role of the encoded tRNA in this bacterium. As an alternative approach, we overexpressed PFL_3991 by cloning it downstream of a constitutive promoter in plasmid pME6010. Overexpression of PFL_3991 significantly increased pyoluteorin production by Pf-5 (Figure 4). These data, in line with the result that optimizing Arg codons in *pltR* promoted the pyoluteorin production (Figure 3B), indicated that biased Arg codon usage regulates pyoluteorin biosynthesis.

### Optimizing the AGA rare codon of *gfp* enhances GFP activity in Pf-5

**Figure 5:**
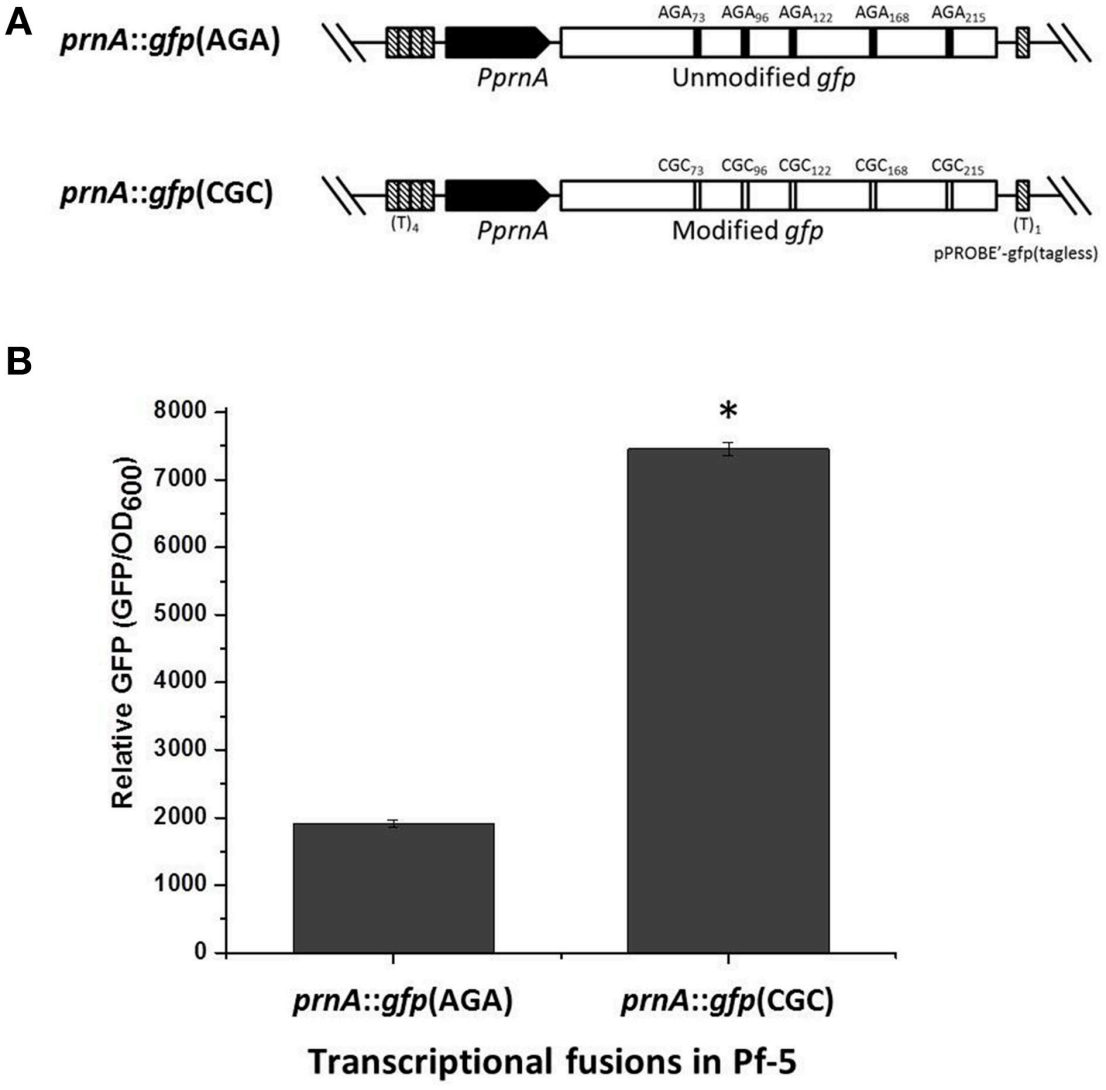
**Substitution of the AGA rare codon of *gfp* with a synonymous preferred codon increased GFP activity in Pf-5**. **(A)** Diagram to show the modification of AGA rare codons in plasmid pPROBE'-gfp(tagless). Only part of the plasmid is shown. The black bars in the above diagram above show the five AGA rare codons in the transcriptional fusion *prnA::gfp*(AGA). The empty bars in the lower diagram show the synonymous codons that were substituted for the AGA codons in *prnA::gfp*(CGC). **(B)** The relative GFP activity of Pf-5 containing the AGA codon-modified transcriptional fusion *prnA::gfp*(CGC), denoted with an asterisk, was significantly higher (student *t*-test, *p* < 0.01) than Pf-5 containing the unmodified *prnA::gfp*(AGA). No obvious growth (OD_600_) difference was observed between strains (data not shown). Values represent the average of four replicates and error bars show the standard deviation.

To further investigate the importance of the AGA codon in gene expression of *P. protegens* Pf-5 in a system independent of pyoluteorin production, we compared GFP activity from a native *gfp*, which has five AGA codons, to that from a modified *gfp*, in which the AGA codons were substituted with the preferred synonymous codon CGC (usage frequency 36.6, Table 2). Both the wild-type *gfp* and the AGA-codon modified *gfp* genes were fused with the promoter of the pyrrolnitrin biosynthesis gene *prnA* (Figure 5A). The GFP activities expressed from the resultant transcriptional fusions *prnA::gfp*(CGC) and *prnA::gfp*(AGA) in wild-type Pf-5 were assayed in 24 h cultures grown in NBGly. As shown in Figure 5B, Pf-5 harboring *prnA::gfp*(CGC) expressed significantly higher levels of GFP fluorescence than the strain harboring *prnA::gfp*(AGA), although both transcriptional fusions were expressed from the same promoter. These results indicated that optimizing the AGA rare codon of *gfp* promoted GFP expression, thereby providing a second line of evidence for the importance of the AGA rare codon in gene expression in *P. protegens* Pf-5.

### Pf-5 derivative strains with a codon-optimized *pltR* overexpress the pyoluteorin biosynthesis gene *pltL*

We have shown that modification of the AGA rare codons of *pltR* and *gfp* into preferred synonymous codons significantly promoted pyoluteorin production (Figure 3), and GFP expression (Figure 5), respectively. Therefore, it is reasonable to propose that the AGA codon optimization increases PltR levels in the bacterial cell, which in turn enhances the transcription of pyoluteorin biosynthesis genes and the production of pyoluteorin. We attempted to test this proposed mechanism directly by quantifying the PltR levels in the bacterial cell but our repeated attempts were not successful due to degradation of PltR in our experiments (data not shown). As an alternative, we evaluated the expression of *pltL* (Figure 2A), which is regulated by the transcriptional regulator PltR (Nowak-Thompson et al., 1999; Yan et al., 2007; Li et al., 2012), as an indirect indicator of PltR activity. To this end, we constructed a plasmid-borne transcriptional fusion of *gfp* to the promoter of *pltL* (*pltL::gfp*) (Figure 3C, inside diagram), and introduced it into Pf-5 as well as derivative strains having codon substitutions in *pltR*. GFP fluorescence level expressed from *pltL::gfp* was significantly higher in LK298, LK364, and LK365, in which AGA rare codons were optimized, than in wild-type Pf-5 (Figure 3C). Similarly, LK362 and LK363 had a higher level of GFP fluorescence from *pltL::gfp* than did wild-type Pf-5. The difference in GFP fluorescence levels of LK361, which has a *pltR* with AGA codons, and Pf-5 was not significant (Figure 3C) although LK361 did produce more pyoluteorin than the wild-type strain (Figure 3A). The increased promoter activity of pyoluteorin biosynthetic gene *pltL* in five of the codon-modified Pf-5 strains is consistent with the higher pyoluteorin production of these strains compared to the wild-type (Figure 3B). These data support the hypothesis that the presence of the rare AGA codon in *pltR* results in reduced levels of PltR, which in turn regulates pyoluteorin production by controlling the transcription of pyoluteorin biosynthetic genes.

### The rare AGA codon is present in genes with diverse predicted functions in the genome of Pf-5

**Figure 6:**
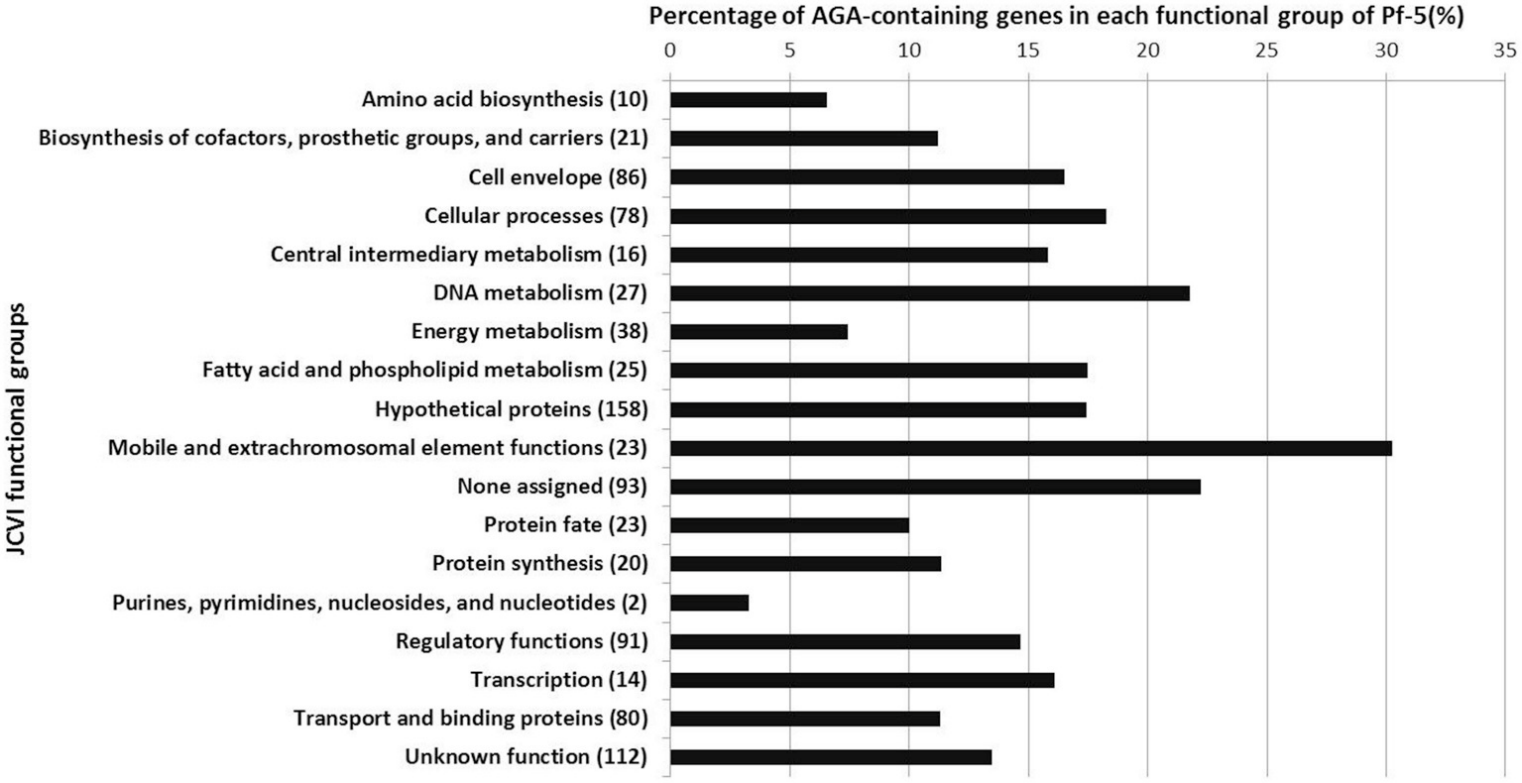
**Functional classification of the 917 AGA-containing genes in the genome of Pf-5**. The percentage of AGA-containing genes in each JCVI functional role category is shown. Numbers in parentheses indicate the total number of AGA-containing genes in each JCVI functional role category for the Pf-5 genome.

The observation that the AGA rare codon regulates pyoluteorin production prompted us to investigate the distribution of the AGA codon in the genome of Pf-5. Strain Pf-5 has 917 AGA-containing genes, which comprise ca. 15% of the protein-coding genes in the genome (Table 2). These 917 AGA-containing genes fall into all of the JCVI functional role categories (Figure 6, Supplemental file 2), indicating that AGA-containing genes collectively have broad functions in the physiology of the bacterial cell. In the genome of Pf-5, the functional groups “Regulatory functions” and “Transcription” have 91 and 14 AGA-containing genes, respectively. Many genes predicted to encode sigma factors, sigma factor-associated regulators, and other regulatory proteins were among the AGA-containing genes within these two functional groups. Examples include *rpoN* (sigma-54, σ^54^) and genes predicted to encode four sigma-54 dependent transcriptional regulators (PFL_1636, 5041, 5309, 5468), five sigma-70 factors (PFL_1373, 2746, 3156, 4041, 5720), and seven GGDEF-containing proteins (PFL_0087, 2458, 3325, 3596, 4322, 4715, 5054). The “Cell envelope” functional group contains 86 AGA-containing genes. This functional group contains large numbers of genes encoding proteins involved in type I or IV pilus formation and polysaccharide synthesis. For example, three AGA-containing genes (PFL_3592, 3593, 3594) encode type I pilus proteins, and six AGA-containing genes (*pilCLNOV*, PFL_5311) encode type IV pilus proteins. In addition, at least 14 AGA-containing genes (*pslDJC, wbpML, lptC*, PFL_0526, 3082, 5099, 5100, 5101, 5102, 5103, 5104) are involved in polysaccharide synthesis. Representatives of the “Cellular processes” functional group include genes encoding catalase KatE and KatG, and the multidrug resistance protein PmpM. Overall, our analyses revealed that the small fraction of genes containing the AGA rare codon participate in diverse functions in *P. protegens* Pf-5, including but not restricted to antibiotic production.

## Discussion

Biased codon usage is known to regulate gene expression and influence cell physiology in bacteria (Quax et al., 2015), but its role in antibiotic production by Gram-negative bacteria remains obscure. In this work, we showed that synonymous modification of codons had a significant effect on pyoluteorin production by the soil bacterium *P. protegens* Pf-5. Our study focused on AGA, the rarest codon used by Pf-5, and PltR, a transcriptional activator of genes for the biosynthesis of the antibiotic pyoluteorin. Substitution of AGA with a common synonymous codon in *pltR* significantly enhanced pyoluteorin production and the promoter activity of a pyoluteorin gene (*pltL*) that is positively regulated by PltR (Figure 3). Also, overexpression of the gene PFL_3991 encoding the cognate tRNA${}_{\text{UCU}}^{\text{Arg}}$ significantly increased pyoluteorin production by *P. protegens* (Figure 4). Taken together, these results support our hypothesis that the rare codon AGA regulates pyoluteorin production in Pf-5.

Of the six codon-modified variants of *pltR* constructed in this study, the variant in LK298, in which 35 types of rare codons were substituted with preferred synonymous codons, conferred much higher pyoluteorin production and *pltL* promoter activity than the other five *pltR* variants, which had fewer substitutions (Figure 3). Possible explanations for the vastly different fold change in antibiotic production and promoter activity between LK298 and other codon-modified strains include the following. (1) There could be a combined influence of the 35 types of rare codons that were substituted in LK298, only a subset of which were substituted in each of the other five strains. In support of this idea, each of the five mutants with one to six substitutions of rare codons exhibited significantly enhanced pyoluteorin production relative to wild-type Pf-5. Therefore, we were not able to eliminate any of the rare codons in *pltR* as irrelevant to pyoluteorin production by this approach, leaving open the possibility that many or all of the rare codons influence production of the antibiotic. While the influence of some rare codons may have been small, the combined influence was substantial, as seen for LK298. (2) In addition to the 23 rare codons that were completed modified in LK298 and in at least one of the five derivative strains (LK361-5), another 12 rare codons that were partially substituted in LK298 may also play a role in the regulation of pyoluteorin production. These 12 rare codons were not substituted with preferred synonymous codons in the modified *pltR* genes present in any of the other five derivative strains tested in this study (Table 3). Further research is needed to investigate the roles of other rare codons in the regulation of pyoluteorin production. (3) The large number of nucleotide differences between the *pltR* of LK298 and the *pltR* of Pf-5 may have altered the structure of the mRNA in a way that enhanced transcript abundance or stability or translational efficiency by a mechanism independent of codon-anticodon interactions. A synonymous codon substitution can alter the secondary structure of mRNA which then influences translation (Bartoszewski et al., 2010). Differences in pyoluteorin production and *pltL* promoter activity between codon-modified strains may be contributed, at least partially, by variations in mRNA structure introduced by nucleotide substitutions.

In the pyoluteorin biosynthesis gene cluster, the AGA rare codon is present not only in *pltR*, but also in six other genes (Figure 2B). Among the AGA-containing genes in the cluster, *pltMR* are required for transcriptional activation of the pyoluteorin structural genes (Nowak-Thompson et al., 1999; Yan et al., 2007; Li et al., 2012; Yan and Loper, unpublished data), *pltLBDE* encode biosynthetic enzymes (Nowak-Thompson et al., 1999; Dorrestein et al., 2005), and *pltZ* encodes a transcriptional regulator controlling the efflux of pyoluteorin from the cell (Huang et al., 2004; Brodhagen et al., 2005). The presence of AGA rare codons in 40% of the genes (Table 2) in the pyoluteorin gene cluster suggests that the bacteria may use the regulatory function of AGA codon as a mechanism to coordinate the regulation, biosynthesis and export of pyoluteorin production. Additionally, we noticed that the AGA rare codon is present in many genes outside of the pyoluteorin gene cluster that influence pyoluteorin production by Pf-5. For example, AGA is present in genes encoding the sigma factor RpoN, which is known to regulate pyoluteorin production by *P. protegens* (Péchy-Tarr et al., 2005). In addition, biosynthesis of pyoluteorin requires the halogenases PltA (Dorrestein et al., 2005) and PltM (Yan and Loper, unpublished data) whose enzyme activities rely on flavin adenine dinucleotide hydroquinone (FADH2), which is produced from flavin adenine dinucleotide (FAD) by flavin reductase. There are seven genes encoding putative flavin reductases in the genome of Pf-5, and none of them are in the pyoluteorin gene cluster (Nowak-Thompson et al., 1999). However, two genes encoding flavin reductases, PFL_3289 and PFL_3609, have an AGA rare codon (Supplemental file 2), and their post-transcriptional regulation by the biased AGA codon usage in Pf-5 could contribute to the coordinated production of pyoluteorin. The presence of AGA codons in the pyoluteorin gene cluster and many other genes associated with pyoluteorin production suggests that the AGA codon may be involved in regulation of pyoluteorin beyond its role in *pltR* described herein.

Compared with more common synonymous codons, rare codons generally are recognized by tRNAs that are at low abundance in the cell, which leads to a slower rate of translation (Ikemura, 1985; Elf et al., 2003; Charles et al., 2006; Chaney and Clark, 2015). The strategy of overexpressing the cognate tRNA has been used to increase translation of mRNAs with rare codons, thereby overcoming biased cognate codon usage in bacteria (Baca and Hol, 2000). In this work, overexpression of tRNA${}_{\text{UCU}}^{\text{Arg}}$ increased pyoluteorin production (Figure 4), which provided a second line of evidence that biased codon usage of AGA regulates pyoluteorin production of Pf-5. The overexpression of tRNA${}_{\text{UCU}}^{\text{Arg}}$ may enhance pyoluteorin production directly through elevated expression of *plt* genes (*pltMRBDEZ*) that contain at least one AGA rare codon (Figure 2B), or indirectly by increasing the expression of AGA-containing genes outside of the *plt* gene cluster that are involved in regulation of pyoluteorin production. Future investigation will be needed to elucidate the regulatory mechanism of overexpression of tRNA${}_{\text{UCU}}^{\text{Arg}}$ on the altered pyoluteorin production of Pf-5.

Our finding that biased AGA codon usage of *pltR* regulates pyoluteorin production of Pf-5 is consistent with the known role of biased codon usage of UUA in antibiotic production by *Streptomyces* spp., but also highlights distinctions between the phenomena in these bacteria. Approximately 2% of the genes in the genomes of *Streptomyces* spp. have the UUA codon (Li et al., 2007) whereas ca. 15% of the genes in the Pf-5 genome have the AGA codon (Table 2). In *S. coelicolor*, nine genes, which comprise 6.2% of the 145 UUA-containing genes in the genome, are involved in the antibiotic biosynthesis (Bentley et al., 2002; Li et al., 2007). In contrast, 24 genes, which comprise only 2.6% of the 917 AGA-containing genes of Pf-5, are involved in the biosynthesis of known antibiotics (Table 2). Other AGA-containing genes have diverse predicted cellular functions, including motility, polysaccharide synthesis, stress response, type II and VI secretion systems (Figure 6, Supplemental file 2). Therefore, the AGA rare codon may play a more general role in the physiology of Pf-5 compared to the relatively focused function of the UUA codon in antibiotic production and development in *Streptomyces* spp. In line with that prediction, mutations in *bldA*, which encodes the only tRNA for the UUA codon of *Streptomyces* spp. (Lawlor et al., 1987), can be selected for and the mutant can persist in laboratory cultures. In contrast, our efforts to delete the tRNA^Arg^ encoding gene PFL_3991 from the chromosome of Pf-5 were unsuccessful, possibly due to an essential role of this tRNA in the bacterium. Additionally, the influence of UUA codon on antibiotic production by *Streptomyces* spp. is primarily through pathway-specific transcriptional regulators encoded by genes containing the UUA codon (Chater and Chandra, 2008). In contrast, the AGA rare codon is present not only in the pathway-specific regulator *pltR*, but also in the structural and export genes in the *plt* gene clusters as well as genes dispersed in the Pf-5 genome that are associated with pyoluteorin production (Figure 2). Thus, biased codon usage has a role in the antibiotic production in both *P. protegens* and *Streptomyces* spp., but these two known examples of codon usage for regulation of antibiotic production have important differences.

In this work, we focused on the AGA rare codons of *pltR* and their roles in pyoluteorin production but our bioinformatic screening suggests that rare codons are likely to influence the production of many antibiotics in Pf-5. AGA is present in biosynthesis gene clusters for toxoflavin, orfamide, rhizoxin, pyrrolnitrin and DAPG, in addition to pyoluteorin (Figure 1, Table 2). For example, *rzxB*, the first gene of the rhizoxin gene cluster, has seven AGA codons. Additionally, both *prnA* and *prnD*, the first and last genes in the pyrrolnitrin gene cluster, have an AGA codon. It would be interesting to investigate the potential role of codon usage in pyrrolnitrin production, as the pyrrolnitrin gene cluster lacks a pathway-specific regulator and little is known about factors controlling production of this antibiotic. In addition to the codon AGA, other rare codons may also play a role in the regulation of antibiotic production by Pf-5. For example, codon UUA, with a usage frequency of 0.8 per 1000 codons in the genome, is the second rarest codon in Pf-5 but is present in *pltR, phlF* and *toxR*, which encode pathway-specific regulators for the production of pyoluteorin, DAPG and toxoflavin, respectively (Nowak-Thompson et al., 1994, 1999; Philmus et al., 2015). The UUA codon was modified to synonymous optimal codons in LK298 and LK364 (Table 3), which have a higher production of pyoluteorin and *pltL* promoter activity than does the wild-type (Figure 3B). Investigations into the regulatory roles of rare codons on antibiotic production by Pf-5 will improve our understanding of codon usage mechanisms and the regulation of bacterial antibiotic production.

Codon usage provides a key mechanism by which microorganisms respond to environmental perturbations (Ling et al., 2015). An example is provided by a recent codon usage analysis in *E. coli*, which showed that rare codons in the yellow fluorescent protein (*yfp*) gene have no obvious impact on the protein synthesis rate in amino acid-rich media, but significantly affect the translation rate under amino acid-starvation conditions (Subramaniam et al., 2013). In the rhizosphere, bacteria like Pf-5 live in dynamic environments influenced by nutrients from root exudates, physical properties of the soil, and interactions with other microbes. The presence of large numbers of rare codons in *pltR* may allow Pf-5 to regulate the production of pyoluteorin, which is known to play a role in microbial interactions (Howell and Stipanovic, 1979; Brodhagen et al., 2004), over the range of fluctuating environmental conditions the bacterium encounters in its natural habitats. We also recognize that AGA and other rare codons are present not only in antibiotic biosynthesis gene clusters, but also many other genes with diverse predicted functions in the genome of Pf-5. Future research on the regulatory roles of rare codons in antibiotic production and the expression of other phenotypes will further advance our knowledge of how *Pseudomonas* spp. coordinate diverse cellular functions with the antibiotic production.

## Author contributions

QY conceived and planned experiments to address hypotheses, carried out the experiments, interpreted results, wrote the first draft of the manuscript. BP conceived the original hypotheses, quantified antibiotic production by bacteria, assisted in manuscript preparation. CH did bioinformatic analyses demonstrating the distribution of rare codons in the Pf-5 genome. MK carried out experiments in the laboratory to derive mutants and extract cultures for antibiotic quantification. JC provided suggestions critical to planning the experiments, and assisted in manuscript preparation. JL led the study in conception of the work, determining the direction of the project, overseeing experimental design, and manuscript preparation.

### Conflict of interest statement

The authors declare that the research was conducted in the absence of any commercial or financial relationships that could be construed as a potential conflict of interest.
