# Supplementary material for: The Rare Codon AGA Is Involved in Regulation of Pyoluteorin Biosynthesis in Pseudomonas protegens Pf-5
Source: Front Microbiol. 2016 Apr 19;7:497. doi: 10.3389/fmicb.2016.00497 (PMC4836200; doi:10.3389/fmicb.2016.00497)

**Supplemental file one containing the following information:**

1. Seven DNA sequences of *pltR* gene of strain Pf-5, LK298, LK361, LK362, LK363, LK364 and LK365.
2. Alignment of the seven *pltR* DNA sequences
3. Alignment of the seven PltR protein sequences
4. Two DNA sequences of *gfp* in the transcriptional fusion of *prnA::gfp*(AGA) and *prnA::gfp*(CGC) (Figure 5A).
5. Alignment of the two *gfp* DNA sequences
6. Alignment of the two GFP protein sequences

1-1) *pltR*-Pf-5 (PFL_2785)

| \| 1 61 121 181 241 301 361 421 481 541 601 661 721 781 841 901 961 1021 \| ATGAAGGCGC TAGGCGTTGT CAATGACATA GACCTTTATA TATCCGTAAC TAAAACGGGT  AGTTTCTCCG AAACAGGAAG ACTACTTGGC ATCCCACCTT CTTCGGTGAT GCGCAGAATC  AATAGCCTCG AAAAAGAACT TGAAACCTGT CTATTCAATC GATCGACAAA ATGCTTGATA  CTTACCGAAA CAGGCCTGCT CTTTCTCGAA CATGCAAAGA ACATCTCGAG ATGCATTGGG  CATGCTAGAT CAGAAGTAAA AGAACACACT GCATCGACCC TTGGCGTTCT CAAGGTTTCA  GCACCCGTGG CCTTTGGACG GCGCCATGTA GCGCCTTTAT TGAGCAGAAT ACTGAACCAT  CATCCCGGTT TGAAAATTGA ATTCTCGCTT AACGACAAAG CTCTCGACCC CAGCATCGAT  AACGTCGATA TCTGTATCAA GCTGGGCATA TTGCCGGACA GTAATCTGAT TCCAACGAAA  CTTGCGGATA TGCGGCGCGT GCTCTGCGCA AGCCCGGAAT ACATCCGGCA ACACGGCTGT  CCGCAAACTC TTGAAGACCT GTACCAACAT GCCTGCCTGA TCCACAGCAC CTGCAGCAAC  TTCTCACTGA CCTGGCAATT CAAGGTCGAT GGCCTGCTCA AGAAGCTCAT GCCCAGCAGC  CGGCTATCGG TCAACAGTTC CGAGTTGCTG GTGGACGGCG CCCTTCAGGG CATAGGAATC  ATCCACGCGC CCACCTGGCT GGTGCATGAA CAGATCGCCA GCGGCCAACT GGTGTCGCTG  CTCGACGAAT ATTGCGAGGC CGATCCGCAA CAGGGAGCGA TCTACGCACT GAGGGCACGC  AGCAGCGTTG TCCCGGCCAA GACCCGCCTG TTCATCAATG AGTTGAAACG CTCGATCGGC  AGCACGCCTT ACTGGGACTT GCCGTTTGAA AAGGAAATAC CGCAGACCCT GGCCACCCTG  CATTTCGATA CCGCAATGCA TTCAGCGCTT TCCAGAGCGA CCACCTTGCA AGACAAGAGC  CAGACATCAT GA \| \| --- \| --- \| |  |
| --- | --- | --- | --- |

1-2) *pltR-*LK298

| 1 61 121 181 241 301 361 421 481 541 601 661 721 781 841 901 961 1021 | ATGAAGGCGC TGGGCGTGGT GAACGACATC GACCTGTACA TCAGCGTGAC CAAGACGGGC  AGCTTCAGCG AGACCGGCCG GCTCCTGGGC ATCCCGCCCA GCAGCGTCAT GCGCCGCATC  AACTCGCTCG AAAAGGAGCT CGAGACCTGC CTGTTCAACC GCTCCACCAA GTGCCTGATC  CTGACCGAGA CGGGCCTGCT GTTCCTGGAA CACGCCAAGA ACATCAGCCG CTGCATCGGC  CACGCCCGGA GCGAAGTCAA GGAACATACC GCGAGCACCC TCGGCGTCCT GAAGGTGTCG  GCGCCGGTCG CGTTCGGCCG TCGCCATGTC GCCCCGCTCC TGAGCCGCAT CCTGAACCAC  CACCCGGGGC TGAAGATCGA ATTCTCGCTG AACGATAAGG CCCTGGACCC GTCGATCGAC  AATGTCGACA TCTGCATCAA GCTCGGCATC CTGCCGGATT CCAACCTCAT CCCGACCAAA  CTGGCCGATA TGCGCCGCGT GCTGTGCGCG TCGCCGGAGT ACATCCGCCA GCATGGCTGC  CCGCAGACCC TCGAGGACCT GTACCAGCAC GCCTGCCTGA TCCACTCGAC CTGCAGCAAC  TTCAGCCTCA CCTGGCAGTT CAAGGTCGAC GGCCTCCTGA AGAAACTGAT GCCGTCCAGC  CGCCTCTCCG TCAACTCCTC CGAACTGCTG GTCGACGGCG CGCTCCAGGG CATCGGCATC  ATCCACGCCC CGACCTGGCT CGTGCATGAG CAGATCGCCA GCGGCCAGCT GGTGAGCCTC  CTCGACGAGT ACTGCGAGGC GGACCCGCAG CAAGGCGCCA TCTACGCCCT CCGCGCGCGT  TCGTCCGTGG TCCCGGCCAA GACCCGGCTC TTCATCAACG AGCTGAAGCG CAGCATCGGC  AGCACCCCCT ACTGGGACCT CCCGTTCGAA AAAGAAATCC CGCAGACCCT GGCCACCCTG  CACTTCGACA CCGCCATGCA CTCCGCCCTG AGCCGCGCCA CCACCCTGCA GGACAAGAGC  CAGACATCAT GA |
| --- | --- |

1-3) *pltR-*LK361

| 1 61 121 181 241 301 361 421 481 541 601 661 721 781 841 901 961 1021 | ATGAAGGCGC TGGGCGTGGT CAATGACATC GACCTGTACA TCTCCGTGAC TAAAACGGGT  AGTTTCTCCG AAACAGGAAG ACTCCTGGGC ATCCCACCTT CTTCGGTGAT GCGCAGAATC  AATAGCCTCG AAAAAGAACT CGAAACCTGT CTGTTCAATC GATCGACAAA ATGCTTGATC  CTGACCGAAA CAGGCCTGCT CTTTCTCGAA CATGCAAAGA ACATCTCGAG ATGCATTGGG  CATGCTAGAT CAGAAGTCAA AGAACACACT GCATCGACCC TCGGCGTCCT CAAGGTGTCA  GCACCCGTGG CCTTTGGACG GCGCCATGTC GCGCCTTTAT TGAGCAGAAT CCTGAACCAT  CATCCCGGTT TGAAAATTGA ATTCTCGCTG AACGACAAAG CTCTCGACCC CAGCATCGAT  AACGTCGATA TCTGTATCAA GCTGGGCATC TTGCCGGACA GTAATCTGAT TCCAACGAAA  CTGGCGGATA TGCGGCGCGT GCTCTGCGCA AGCCCGGAAT ACATCCGGCA ACACGGCTGT  CCGCAAACTC TCGAAGACCT GTACCAACAT GCCTGCCTGA TCCACAGCAC CTGCAGCAAC  TTCTCACTGA CCTGGCAATT CAAGGTCGAT GGCCTGCTCA AGAAGCTCAT GCCCAGCAGC  CGGCTCTCGG TCAACAGTTC CGAGTTGCTG GTGGACGGCG CCCTCCAGGG CATCGGAATC  ATCCACGCGC CCACCTGGCT GGTGCATGAA CAGATCGCCA GCGGCCAACT GGTGTCGCTG  CTCGACGAAT ACTGCGAGGC CGATCCGCAA CAGGGAGCGA TCTACGCACT GAGGGCACGC  AGCAGCGTGG TCCCGGCCAA GACCCGCCTG TTCATCAATG AGTTGAAACG CTCGATCGGC  AGCACGCCTT ACTGGGACTT GCCGTTTGAA AAGGAAATCC CGCAGACCCT GGCCACCCTG  CATTTCGATA CCGCAATGCA TTCAGCGCTG TCCAGAGCGA CCACCTTGCA AGACAAGAGC  CAGACATCAT GA |
| --- | --- |

1-4) *pltR-*LK362

| 1 61 121 181 241 301 361 421 481 541 601 661 721 781 841 901 961 1021 | ATGAAGGCGC TAGGCGTTGT CAATGACATA GACCTTTATA TATCCGTAAC CAAAACGGGC  AGTTTCTCCG AAACAGGCAG ACTACTTGGC ATCCCGCCCT CTTCGGTGAT GCGCAGAATC  AATAGCCTCG AAAAAGAACT TGAAACCTGT CTATTCAATC GATCGACAAA ATGCTTGATA  CTTACCGAAA CAGGCCTGCT CTTTCTCGAA CATGCAAAGA ACATCTCGAG ATGCATTGGG  CATGCCAGAT CAGAAGTAAA AGAACACACC GCATCGACCC TTGGCGTTCT CAAGGTTTCA  GCACCCGTGG CCTTTGGCCG GCGCCATGTA GCGCCGTTAT TGAGCAGAAT ACTGAACCAT  CATCCCGGGT TGAAAATTGA ATTCTCGCTT AACGACAAAG CCCTCGACCC CAGCATCGAT  AACGTCGATA TCTGTATCAA GCTGGGCATA TTGCCGGACA GTAATCTGAT TCCGACGAAA  CTTGCGGATA TGCGGCGCGT GCTCTGCGCA AGCCCGGAAT ACATCCGGCA ACACGGCTGT  CCGCAAACCC TTGAAGACCT GTACCAACAT GCCTGCCTGA TCCACAGCAC CTGCAGCAAC  TTCTCACTGA CCTGGCAATT CAAGGTCGAT GGCCTGCTCA AGAAGCTCAT GCCCAGCAGC  CGGCTATCGG TCAACAGTTC CGAGTTGCTG GTGGACGGCG CCCTTCAGGG CATAGGCATC  ATCCACGCGC CCACCTGGCT GGTGCATGAA CAGATCGCCA GCGGCCAACT GGTGTCGCTG  CTCGACGAAT ATTGCGAGGC CGATCCGCAA CAGGGCGCGA TCTACGCACT GAGGGCACGC  AGCAGCGTTG TCCCGGCCAA GACCCGCCTG TTCATCAATG AGTTGAAACG CTCGATCGGC  AGCACGCCCT ACTGGGACTT GCCGTTTGAA AAGGAAATAC CGCAGACCCT GGCCACCCTG  CATTTCGATA CCGCAATGCA TTCAGCGCTT TCCAGAGCGA CCACCTTGCA AGACAAGAGC  CAGACATCAT GA |
| --- | --- |

1-5) *pltR-*LK363

| 1 61 121 181 241 301 361 421 481 541 601 661 721 781 841 901 961 1021 | ATGAAGGCGC TAGGCGTTGT CAATGACATA GACCTTTATA TATCCGTAAC TAAAACGGGT  AGTTTCTCCG AAACAGGAAG ACTACTTGGC ATCCCACCTA GCTCGGTGAT GCGCAGAATC  AATAGCCTCG AAAAAGAACT TGAAACCTGC CTATTCAATC GCTCGACAAA ATGCCTGATA  CTTACCGAAA CAGGCCTGCT CTTCCTCGAA CATGCCAAGA ACATCTCGAG ATGCATTGGG  CATGCTAGAT CAGAAGTAAA AGAACACACT GCGTCGACCC TTGGCGTTCT CAAGGTTTCA  GCGCCCGTGG CCTTCGGACG GCGCCATGTA GCGCCTTTAC TGAGCAGAAT ACTGAACCAT  CATCCCGGTC TGAAAATTGA ATTCTCGCTT AACGACAAAG CTCTCGACCC CAGCATCGAT  AACGTCGATA TCTGCATCAA GCTGGGCATA CTGCCGGACA GTAATCTGAT TCCAACGAAA  CTTGCGGATA TGCGGCGCGT GCTCTGCGCG AGCCCGGAAT ACATCCGGCA ACACGGCTGC  CCGCAAACTC TTGAAGACCT GTACCAACAT GCCTGCCTGA TCCACAGCAC CTGCAGCAAC  TTCTCACTGA CCTGGCAATT CAAGGTCGAT GGCCTGCTCA AGAAGCTCAT GCCCAGCAGC  CGGCTATCGG TCAACAGTTC CGAGCTGCTG GTGGACGGCG CCCTTCAGGG CATAGGAATC  ATCCACGCGC CCACCTGGCT GGTGCATGAA CAGATCGCCA GCGGCCAACT GGTGTCGCTG  CTCGACGAAT ATTGCGAGGC CGATCCGCAA CAGGGAGCGA TCTACGCCCT GAGGGCGCGC  AGCAGCGTTG TCCCGGCCAA GACCCGCCTG TTCATCAATG AGCTGAAACG CTCGATCGGC  AGCACGCCTT ACTGGGACCT CCCGTTCGAA AAGGAAATAC CGCAGACCCT GGCCACCCTG  CATTTCGATA CCGCCATGCA TTCAGCGCTT TCCAGAGCGA CCACCCTGCA AGACAAGAGC  CAGACATCAT GA |
| --- | --- |

1-6) *pltR-*LK364

| 1 61 121 181 241 301 361 421 481 541 601 661 721 781 841 901 961 1021 | ATGAAGGCGC TAGGCGTTGT CAATGACATA GACCTTTATA TATCCGTAAC TAAAACGGGT  AGCTTCTCCG AAACAGGACG GCTACTTGGC ATCCCACCTT CTTCGGTGAT GCGCCGCATC  AATAGCCTCG AAAAAGAACT TGAAACCTGT CTATTCAATC GATCGACAAA ATGCTTGATA  CTTACCGAAA CAGGCCTGCT CTTTCTCGAA CATGCAAAGA ACATCTCGCG CTGCATCGGG  CATGCTCGGT CAGAAGTAAA AGAACACACT GCATCGACCC TTGGCGTTCT CAAGGTTTCA  GCACCCGTGG CCTTTGGACG GCGCCATGTA GCGCCTCTCT TGAGCCGCAT ACTGAACCAT  CATCCCGGTT TGAAAATCGA ATTCTCGCTT AACGACAAAG CTCTCGACCC CAGCATCGAT  AACGTCGATA TCTGTATCAA GCTGGGCATA TTGCCGGACT CCAATCTGAT CCCAACGAAA  CTTGCGGATA TGCGGCGCGT GCTCTGCGCA AGCCCGGAAT ACATCCGGCA ACACGGCTGT  CCGCAAACTC TTGAAGACCT GTACCAACAT GCCTGCCTGA TCCACAGCAC CTGCAGCAAC  TTCTCACTGA CCTGGCAATT CAAGGTCGAT GGCCTGCTCA AGAAGCTCAT GCCCAGCAGC  CGGCTATCGG TCAACTCCTC CGAGTTGCTG GTGGACGGCG CCCTTCAGGG CATAGGAATC  ATCCACGCGC CCACCTGGCT GGTGCATGAA CAGATCGCCA GCGGCCAACT GGTGTCGCTG  CTCGACGAAT ATTGCGAGGC CGATCCGCAA CAGGGAGCGA TCTACGCACT GCGCGCACGC  AGCAGCGTTG TCCCGGCCAA GACCCGCCTG TTCATCAATG AGTTGAAACG CTCGATCGGC  AGCACGCCTT ACTGGGACTT GCCGTTTGAA AAGGAAATAC CGCAGACCCT GGCCACCCTG  CATTTCGATA CCGCAATGCA TTCAGCGCTT TCCCGCGCGA CCACCTTGCA AGACAAGAGC  CAGACATCAT GA |
| --- | --- |

1-7) *pltR-*LK365

| 1 61 121 181 241 301 361 421 481 541 601 661 721 781 841 901 961 1021 | ATGAAGGCGC TAGGCGTTGT CAATGACATA GACCTTTATA TATCCGTAAC TAAAACGGGT  AGTTTCTCCG AAACAGGACG GCTACTTGGC ATCCCACCTT CTTCGGTGAT GCGCCGCATC  AATAGCCTCG AAAAAGAACT TGAAACCTGT CTATTCAATC GATCGACAAA ATGCTTGATA  CTTACCGAAA CAGGCCTGCT CTTTCTCGAA CATGCAAAGA ACATCTCGCG CTGCATTGGG  CATGCTCGGT CAGAAGTAAA AGAACACACT GCATCGACCC TTGGCGTTCT CAAGGTTTCA  GCACCCGTGG CCTTTGGACG GCGCCATGTA GCGCCTTTAT TGAGCCGCAT ACTGAACCAT  CATCCCGGTT TGAAAATTGA ATTCTCGCTT AACGACAAAG CTCTCGACCC CAGCATCGAT  AACGTCGATA TCTGTATCAA GCTGGGCATA TTGCCGGACA GTAATCTGAT TCCAACGAAA  CTTGCGGATA TGCGGCGCGT GCTCTGCGCA AGCCCGGAAT ACATCCGGCA ACACGGCTGT  CCGCAAACTC TTGAAGACCT GTACCAACAT GCCTGCCTGA TCCACAGCAC CTGCAGCAAC  TTCTCACTGA CCTGGCAATT CAAGGTCGAT GGCCTGCTCA AGAAGCTCAT GCCCAGCAGC  CGGCTATCGG TCAACAGTTC CGAGTTGCTG GTGGACGGCG CCCTTCAGGG CATAGGAATC  ATCCACGCGC CCACCTGGCT GGTGCATGAA CAGATCGCCA GCGGCCAACT GGTGTCGCTG  CTCGACGAAT ATTGCGAGGC CGATCCGCAA CAGGGAGCGA TCTACGCACT GAGGGCACGC  AGCAGCGTTG TCCCGGCCAA GACCCGCCTG TTCATCAATG AGTTGAAACG CTCGATCGGC  AGCACGCCTT ACTGGGACTT GCCGTTTGAA AAGGAAATAC CGCAGACCCT GGCCACCCTG  CATTTCGATA CCGCAATGCA TTCAGCGCTT TCCCGCGCGA CCACCTTGCA AGACAAGAGC  CAGACATCAT GA |
| --- | --- |

2) Alignment of the seven *pltR* DNA sequences


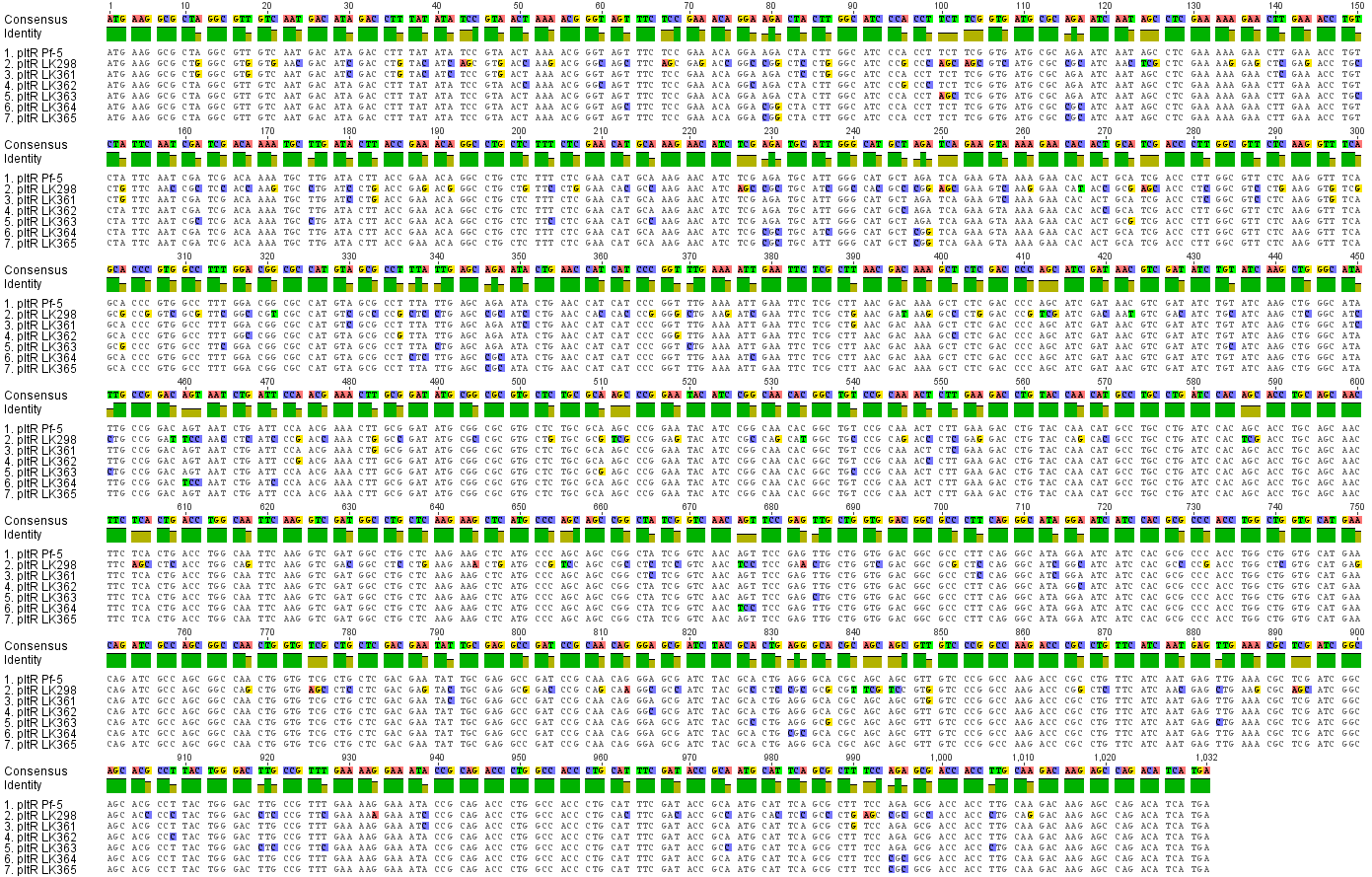


3) Alignment of the seven PltR protein sequences


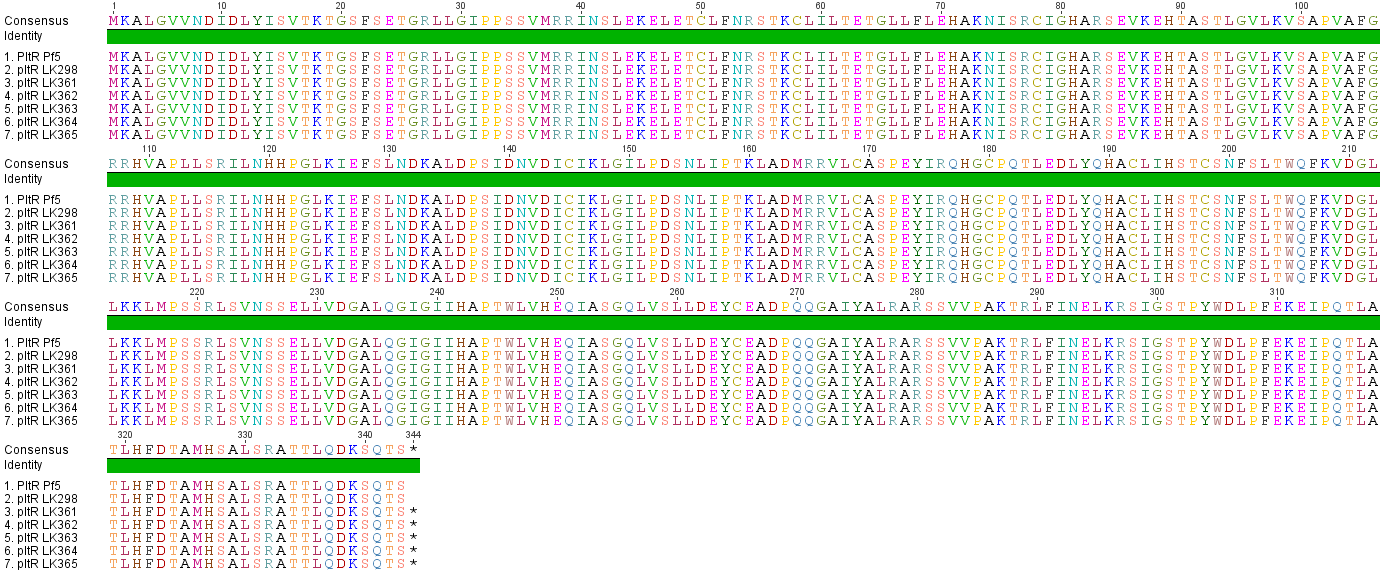


4-1) *gfp-* *prnA::gfp*(AGA)

| 1 61 121 181 241 301 361 421 481 541 601 661 | ATGAGTAAAG GAGAAGAACT TTTCACTGGA GTTGTCCCAA TTCTTGTTGA ATTAGATGGT  GATGTTAATG GGCACAAATT TTCTGTCAGT GGAGAGGGTG AAGGTGATGC AACATACGGA  AAACTTACCC TTAAATTTAT TTGCACTACT GGAAAACTAC CTGTTCCATG GCCAACACTT  GTCACTACTT TGACTTATGG TGTTCAATGC TTTTCAAGAT ACCCAGATCA TATGAAACGG  CATGACTTTT TCAAGAGTGC CATGCCCGAA GGTTATGTAC AGGAAAGAAC TATATTTTTC  AAAGATGACG GGAACTACAA GACACGTGCT GAAGTCAAGT TTGAAGGTGA TACCCTTGTT  AATAGAATCG AGTTAAAAGG TATTGATTTT AAAGAAGATG GAAACATTCT TGGACACAAA  TTGGAATACA ACTATAACTC ACACAATGTA TACATCATGG CAGACAAACA AAAGAATGGA  ATCAAAGTTA ACTTCAAAAT TAGACACAAC ATTGAAGATG GAAGCGTTCA ACTAGCAGAC  CATTATCAAC AAAATACTCC AATTGGCGAT GGCCCTGTCC TTTTACCAGA CAACCATTAC  CTGTCCACAC AATCTGCCCT TTCGAAAGAT CCCAACGAAA AGAGAGACCA CATGGTCCTT  CTTGAGTTTG TAACAGCTGC TGGGATTACA CATGGCATGG ATGAACTATA CAAATAA |
| --- | --- |

4-2) *gfp-* *prnA::gfp*(CGC)

| 1 61 121 181 241 301 361 421 481 541 601 661 | ATGAGTAAAG GAGAAGAACT TTTCACTGGA GTTGTCCCAA TTCTTGTTGA ATTAGATGGT  GATGTTAATG GGCACAAATT TTCTGTCAGT GGAGAGGGTG AAGGTGATGC AACATACGGA  AAACTTACCC TTAAATTTAT TTGCACTACT GGAAAACTAC CTGTTCCATG GCCAACACTT  GTCACTACTT TGACTTATGG TGTTCAATGC TTTTCACGCT ACCCAGATCA TATGAAACGG  CATGACTTTT TCAAGAGTGC CATGCCCGAA GGTTATGTAC AGGAACGCAC TATATTTTTC  AAAGATGACG GGAACTACAA GACACGTGCT GAAGTCAAGT TTGAAGGTGA TACCCTTGTT  AATCGCATCG AGTTAAAAGG TATTGATTTT AAAGAAGATG GAAACATTCT TGGACACAAA  TTGGAATACA ACTATAACTC ACACAATGTA TACATCATGG CAGACAAACA AAAGAATGGA  ATCAAAGTTA ACTTCAAAAT TCGCCACAAC ATTGAAGATG GAAGCGTTCA ACTAGCAGAC  CATTATCAAC AAAATACTCC AATTGGCGAT GGCCCTGTCC TTTTACCAGA CAACCATTAC  CTGTCCACAC AATCTGCCCT TTCGAAAGAT CCCAACGAAA AGCGCGACCA CATGGTCCTT  CTTGAGTTTG TAACAGCTGC TGGGATTACA CATGGCATGG ATGAACTATA CAAATAA |
| --- | --- |

5) Alignment of the two *gfp* DNA sequences


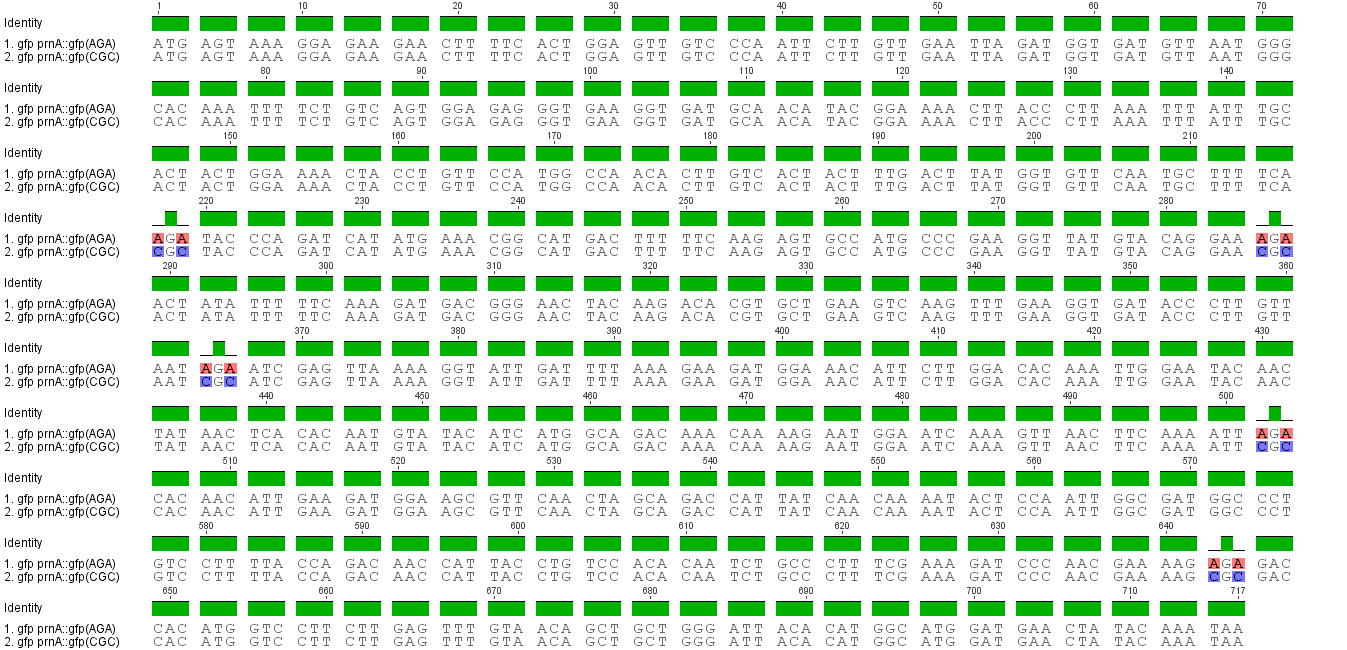


6) Alignment of the two GFP protein sequences


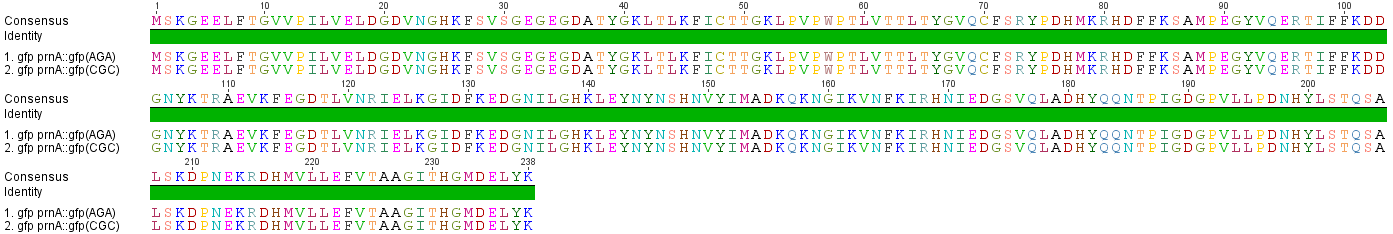

Supplement: Supplementary file 1 [file DataSheet1.DOCX]
